# Multifunctional synthetic nano-chaperone for peptide folding and intracellular delivery

**DOI:** 10.1038/s41467-022-32268-2

**Published:** 2022-08-05

**Authors:** Il-Soo Park, Seongchan Kim, Yeajee Yim, Ginam Park, Jinahn Choi, Cheolhee Won, Dal-Hee Min

**Affiliations:** 1 Department of Chemistry, Seoul National University, Seoul, 08826 Republic of Korea; 2 Biomaterials Research Center, Biomedical Research Division, Korea Institute of Science and Technology (KIST), Seoul, 02792 Republic of Korea; 3 Institute of Biotherapeutics Convergence Technology, Lemonex Inc, Seoul, 06683 Republic of Korea

**Keywords:** Peptide delivery, Drug delivery, Peptides, Nanoparticles

## Abstract

Artificial, synthetic chaperones have attracted much attention in biomedical research due to their ability to control the folding of proteins and peptides. Here, we report bio-inspired multifunctional porous nanoparticles to modulate proper folding and intracellular delivery of therapeutic α-helical peptide. The *S*ynthetic *N*ano-*C*haperone for *P*eptide (SNCP) based on porous nanoparticles provides an internal hydrophobic environment which contributes in stabilizing secondary structure of encapsulated α-helical peptides due to the hydrophobic internal environments. In addition, SNCP with optimized inner surface modification not only improves thermal stability for α-helical peptide but also supports the peptide stapling methods in situ, serving as a nanoreactor. Then, SNCP subsequently delivers the stabilized therapeutic α-helical peptides into cancer cells, resulting in high therapeutic efficacy. SNCP improves cellular uptake and bioavailability of the anti-cancer peptide, so the cancer growth is effectively inhibited in vivo. These data indicate that the bio-inspired SNCP system combining nanoreactor and delivery carrier could provide a strategy to expedite the development of peptide therapeutics by overcoming existing drawbacks of α-helical peptides as drug candidates.

## Abstract (web summary)

Molecular chaperones play an important part in protein folding and delivery in nature. Here, the authors report on the creation of a synthetic chaperone to control the folding of therapeutic peptides from random coil to alpha helix and demonstrate enhanced therapeutic potential in an in vivo tumour model.

## Introduction

Molecular chaperones play important roles in a living system to control protein folding/unfolding and guide it to native structure from partially folded states. With hydrophobic segments or an internal space, the chaperones not only trap unfolded initial proteins or intermediates but also prevent an irreversible aggregation of proteins during the refolding process^1–3^. In many cases, proteins and peptides manufactured from *Escherichia coli* or chemical synthesis do not show enough biological activities in biomedical applications mostly due to undesirable aggregation, poor solubility, or inactive conformation^4^. To solve these problems, artificial biological systems emulating molecular chaperones have attracted lots of attention in the field of biotechnology and biomedicine.

Therapeutic α-helical peptides have gradually emerged as attractive drug candidates for treatment of cancer and diseases. However, they have intrinsic weakness, including poor conformational stability and short half-life by proteolytic degradation and fast clearance^5^. To overcome these drawbacks, various strategies have been suggested including peptide stapling methods that fix α-helical structure of peptides through covalent bond formation^6–13^. Nevertheless, pragmatic challenges still remain for practical biological and medical applications of α-helical peptides due to insufficient cellular uptake and instability in vivo^6^.

In this regard, nanomaterials harnessing various components such as cyclodextrins^14^, polymers^15,16^, metallic nanoparticles^17,18^, silica^19,20^ and self-assembled nanostructures^21–23^ have been developed as artificial molecular chaperones. Among them, porous nanoparticles have shown great potential in delivering bio-macromolecules such as plasmid DNA^24^, siRNA^25–27^ and proteins^28^ to target cells due to tunable pore size, large surface area, and flexible surface modification^29,30^. Therefore, porous nanoparticles are regarded as one of the most promising delivery vehicles for biomedical applications and these previous studies have inspired us to develop a synthetic chaperone system based on porous nanoparticles suitable for assisting proper folding of therapeutic α-helical peptides and for intracellular peptide delivery vehicle at the same time.

**Fig. 1:**
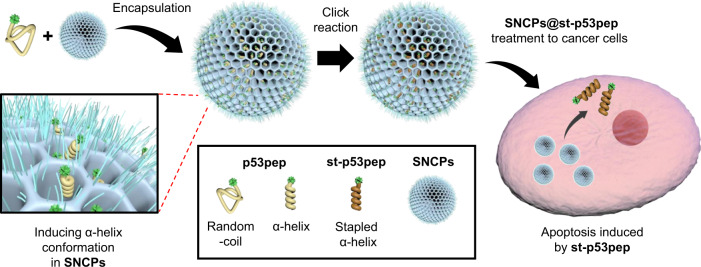
Schematic representation of synthetic nano-chaperones for peptide (SNCP) based on porous nanoparticles. After encapsulation of p53pep, structural stabilization of p53pep is induced from a random-coil structure to an α-helical structure. In situ click reaction for peptide stapling then proceeds while p53pep is loaded inside SNCP to make the α-helical structure of p53pep irreversible. Finally, direct cellular delivery of st-p53pep using SNCP results in p53 mediated apoptosis of cancer cells.

Here, we demonstrate a smart integrated system, designated as synthetic nano-chaperone for peptide (SNCP) as a nanoreactor for engineering chemically stapled α-helical peptide with high stability as well as a delivery carrier for eliciting efficient tumor control via direct intracellular delivery of the therapeutic anti-cancer peptide in its bioactive conformation (Fig. 1). We reveal that a model peptide is successfully transformed from a random-coil structure to an α-helical structure inside pores of SNCP. In addition, in situ peptide stapling is employed sequentially via click reaction to ensure irreversible and thermally stable α-helical structure of the peptide without aggregation. For optimization of SNCP, we also address how the internal environment of porous nanoparticles affects secondary structures of peptides in detail. Then, we successfully demonstrate therapeutic potential of the SNCP system by directly treating the stabilized therapeutic peptide in the SNCP to cancer cells and murine tumor model and showing efficient induction of apoptosis of cancer cells in vitro and tumor growth inhibition in vivo.

## Results

### Design and characterization of SNCPs

**Fig. 2:**
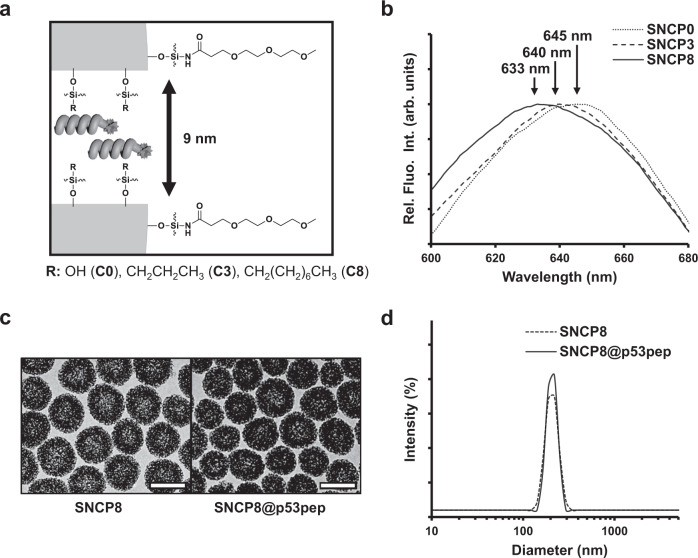
Design of SNCPs and characterization. **a** Illustration of SNCP design. A TEG moiety was conjugated at the outer surface. Hydroxyl (SNCP0), propyl (SNCP3), and octyl (SNCP8) moieties were introduced on the inner surface. **b** Fluorescence spectra of Nile red encapsulated in SNCPs (λ_ex_ = 510 nm). **c** Transmission electron microscopy (TEM) images of SNCP8 and SNCP8@p53pep (scale bar, 200 nm). **d** Dynamic light scattering (DLS) data of SNCP8 and SNCP8@p53pep.

To apply our artificial chaperone system, we selected a synthetic p53 peptide (p53pep) as a model therapeutic peptide. It was derived from a 15-residue α-helical transactivation domain of transcription factor p53 protein which induces apoptosis in response to cellular stress. This α-helical domain binds to a deep hydrophobic cleft of MDM2 oncoprotein which regulates p53 protein levels as a cellular inhibitor^31^. Inspired by the previous study about the stapled p53 peptide, we designed a peptide presenting azido and alkynyl groups at the *i* and *i* + 7 position for peptide stapling via click reaction, avoiding critical MDM2-binding residues^10^ (Supplementary Fig. 1a, b). Fluorescein amidites (FAM) dye with di-glycine linker was conjugated at the N-terminus of p53pep for its visualization. To construct a nanoreactor for SNCPs, we prepared mesoporous silica nanoparticles according to previously reported method^25^. Triethylene glycol moiety (MeO-PEG3-COOH, TEG) was introduced further on the outer surface of SNCPs that improved solubility in aqueous conditions. Thereon, we applied further to hydroxyl (SNCP0), propyl (SNCP3) and octyl (SNCP8) group on the inner surface in SNCPs to discover an appropriate internal environment for stabilizing α-helical structure of p53pep (Fig. 2a). As shown in Fourier-transform infrared (FT-IR) spectra of SNCPs, amide I band from covalent attachment site of TEG on the outer surface, and C-H stretching band from aliphatic chain moieties on the inner surface were observed, respectively (Supplementary Fig. 2). The results show that aliphatic functional groups were introduced successfully on the inner surface of SNCPs. The data from nitrogen sorption analysis showed that the prepared SNCPs possessed mean pore size of below ca.13 nm and Brunauer–Emmett–Teller specific surface area of ca. 330 m^2^/g (Supplementary Fig. 3).

To investigate whether introduction of aliphatic chain moieties on the inner surface of SNCPs enables internal environment of SNCPs to be more hydrophobic, we performed a Nile red assay to compare polarity of internal environment for each SNCP^32^. A spectral blue shift in the fluorescence emission spectrum of encapsulated Nile red in SNCPs was observed as the length of aliphatic chain increases on the inner surface of SNCPs (Fig. 2b). Moreover, the SNCPs modified with aliphatic chain exhibited higher loading capacity for p53pep compared to SNCP0 (Supplementary Fig. 3). These results indicate that the introduction of aliphatic chain functional groups on the inner surface of SNCPs increased hydrophobicity of its inner environment, which could affect interaction between the encapsulated peptide and SNCPs.

Encouraged by the results of successful surface modification and characteristics for SNCPs, we prepared the p53pep encapsulated SNCPs for following functional studies by simple stirring the mixture of p53pep and SNCP in the aqueous condition. Transmission electron microscopy (TEM) images and dynamic light scattering (DLS) data revealed similar size distribution of SNCP with or without encapsulation of the peptide (Fig. 2c, d and Supplementary Fig. 4). These results demonstrate that p53pep was successfully encapsulated in the inner cavity of SNCPs.

### Controlling α-helical conformation and stability of p53pep by SNCPs

**Fig. 3:**
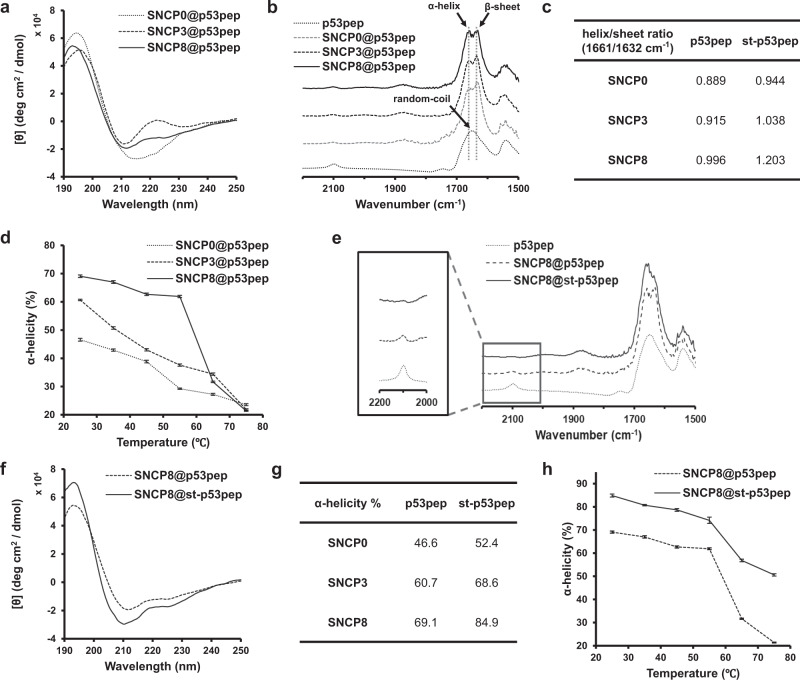
Peptide secondary structure analysis. **a** Circular dichroism (CD) spectra and **b** Fourier-transform infrared (FT-IR) spectra of p53pep in SNCPs. **c** Helix/sheet ratio of p53pep in SNCPs calculated based on the FT-IR spectrum. **d** α-Helicity of p53pep encapsulated in SNCPs with increasing temperature (*N* = 3; mean ± SD). **e** FT-IR spectra and **f** CD spectra of st-p53pep in SNCP8. For FT-IR spectra, the region near azide stretching band was shown in the enlarged image. **g** α-Helicity of p53pep and st-p53pep in SNCPs. **h** α-Helicity of p53pep and st-p53pep in SNCP8 with increasing temperature (*N* = 3; mean ± SD).

As shown in circular dichroism (CD) spectrum (Supplementary Fig. 5), p53pep possessed a random-coil structure in aqueous solution and a typical α-helical structure in 30% 2, 2, 2-trifluoroethanol solution, which suggests that the polarity of surrounding environment for the peptide has a crucial role in controlling its secondary structure^33^. To identify the regulation of secondary structure of p53pep in various inner environment of SNCPs, we prepared three kinds of the p53pep loaded SNCPs (SNCPs@p53pep) complex by using SNCP0, SNCP3 and SNCP8. As shown in CD spectra of SNCPs@p53pep at room temperature, p53pep formed different secondary structures depending on the inner surface environment of SNCPs (Fig. 3a). Especially, CD spectrum of SNCP8@p53pep showed two negative peaks of typical α-helix at 211 and 225 nm that red-shifted from peaks at 208 and 222 nm^34^. On the other hand, CD spectra of SNCP0@p53pep and SNCP3@p53pep showed changes to more irregular structures compared with that of SNCP8@p53pep. The β-sheet structure in SNCP0 could be due to the intermolecular hydrophobic interaction between p53pep as the interaction between the hydrophobic p53pep and the inner nanoparticle surface is not highly preferred in SNCP0. Thus, this result indicates that the hydrophobic environment in SNCP8 more favorably facilitates the encapsulated p53pep to form α-helical structure compared to SNCP0 and SNCP3.

To further investigate the structural conformation of p53pep encapsulated inside the SNCPs, FT-IR experiments for SNCPs@p53pep were performed. FT-IR spectra showed changes in amide I band (between 1600 and 1700 cm^−1^), which is directly related to peptide backbone conformation^35^ (Fig. 3b). The p53pep itself showed one peak of amide I band at 1648 cm^−1^, corresponding to random-coil structures. However, the FT-IR spectrum of p53pep encapsulated in SNCPs exhibited split two peaks which could be assigned to α-helix (1658 cm^−1^) and β-sheet components (1632 cm^−1^), respectively. Moreover, based on the semi-quantitative determination of intensity ratio (helix/sheet ratio) at 1658/1632 cm^−1^, SNCP8 showed the highest ratio of 0.996 compared to those of SNCP0 and SNCP3 (Fig. 3c). Taken together, these results indicate that the structural conformation of α-helical peptide was finely controlled by the hydrophobicity of inner surface inside SNCPs.

Next, the thermal stability of α-helical peptides encapsulated in SNCPs was investigated to evaluate the protective and shielding capability of SNCPs as a molecular chaperone which prevent denaturation of proteins that could be induced by high temperature^36^. The random-coil structure of p53pep itself in aqueous solution showed high thermodynamic stability without a conformational change in various temperature from 25 °C to 75 °C. However, the unstable α-helical conformation was observed in SNCPs@p53pep as temperature increases (Supplementary Fig. 6). To analyze the structural change of p53pep inside SNCPs in detail, the α-helicity of p53pep was determined by using the CONTIN program method^37,38^. Although α-helicity of the encapsulated p53pep decreased as temperature increased, the p53pep loaded into SNCP8 exhibited highest α-helicity up to 55 °C among the tested SNCPs (Fig. 3d). Considering that SNCP0 and SNCP3 showed little contribution to the thermal stability of p53pep relatively, we suggest that hydrophobic interaction between the peptide and the inner surface of SNCPs plays a critical role in improving thermal stability of α-helical peptide.

### ‘Click’ peptide stapling for p53pep assisted by SNCPs

To introduce the irreversible α-helical structure of p53pep even after released from the SNCPs, we introduced the click peptide stapling method using copper(I)-catalyzed azide-alkyne cycloaddition (CuAAC) reaction. The method is simple, cost-effective, and low cytotoxic compared to the ruthenium-based ring-closing olefin metathesis known as the most notable stapling method^39^. The click reaction was conducted in the presence of CuSO_4_ and ascorbic acid in aqueous solution with stirring at 50 °C for overnight. To ensure the advantage of SNCP encapsulation of p53pep and in situ peptide stapling strategy, we calculated the click stapling yield of p53pep by assigning the molecular weight peak of p53pep at the matrix-assisted laser desorption ionization time-of-flight mass spectrometry (MALDI-TOF MS) spectrum after high performance liquid chromatography (HPLC) analysis in various conditions. Since the p53pep is insoluble in water, an organic solvent was used to prepare the st-p53pep without SNCP. The stapling yield for st-p53pep was under 40% in tetrahydrofuran (THF) 50% aqueous solution, while the stapling yield was over 85% in case of the pre-encapsulated p53pep in SNCP in aqueous solution (Supplementary Fig. 7). The result strongly supports the high click reaction efficiency by stabilizing the helical conformation of p53pep inside the hydrophobic cavity of SNCP. Under the employed reaction condition, click reaction was successfully carried out without notable disruption of α-helical structure of p53pep. The stapled peptide products prepared by click reaction (st-p53pep) inside SNCPs (SNCPs@st-p53pep) were confirmed by the reduction of azide stretching band (2105 cm^−1^) compared to free p53pep and SNCPs@p53pep in FT-IR spectrum (Fig. 3e, and Supplementary Fig. 8). The results indicate that st-p53pep was successfully formed by click reaction regardless of hydrophobicity and functional groups on the inner surface of SNCPs^40^.

CD spectra showed the significantly increased molar ellipticity and α-helicity in SNCPs@st-p53pep compared to those of SNCPs@p53pep (Fig. 3f, g and Supplementary Fig 9). In addition, FT-IR spectra of SNCPs@st-p53pep showed increasing helix/sheet ratio as hydrophobicity increases inside SNCPs (Fig. 3c, e, Supplementary Fig. 8). These results demonstrate that the reduced conformational freedom by triazole bond formation strengthens the secondary structure of α-helical peptide. Interestingly, st-p53pep inside SNCP8 showed the highest stabilization of α-helical conformation compared to the others. The secondary structure of stapled peptide inside SNCPs is strongly influenced by the internal environment of SNCPs due to the reduction of conformational entropy^41,42^. Moreover, α-helicity of st-p53pep inside SNCP8 was about 75% at 55 °C and moderately decreased to 50% at 75 °C, suggesting highly improved thermal stability compared to p53pep, which showed α-helicity of only 60 and 20% at 55 and 75 °C, respectively (Fig. 3h and Supplementary Fig. 10). Compared to the st-p53pep@SNCP8 and p53pep@SNCP8, the st-p53pep exhibited the lower α-helicity (34.6%) below 55 °C due to the aggregation with poor water solubility (Supplementary Fig. 11)^43,44^. Furthermore, compared to the SNCP8@st-p53pep, the extracted st-p53pep from SNCP8@st-p53pep showed the lower α-helicity (34.6%) with increased unordered character while recovering the α-helicity (73.4%) after re-encapsulation (Supplementary Fig. 12). Considering that the poor solubility of p53pep may cause unstable peptide structure due to the aggregation in aqueous conditions^43,44^, SNCP encapsulation induces the refolding and maintains the α-helix conformation by isolating the stapled peptide from external environment. This result suggests that the SNCP8 chaperone increases the structure and thermal stability of peptides according to the hydrophobicity of the inner pore surface. Taken together, we chose SNCP8 as the most suitable molecular nanoreactor for the high click reaction efficiency by stabilizing the helical conformation of p53pep and used it for further studies in vitro and in vivo.

### In vitro functional study of st-p53pep

**Fig. 4:**
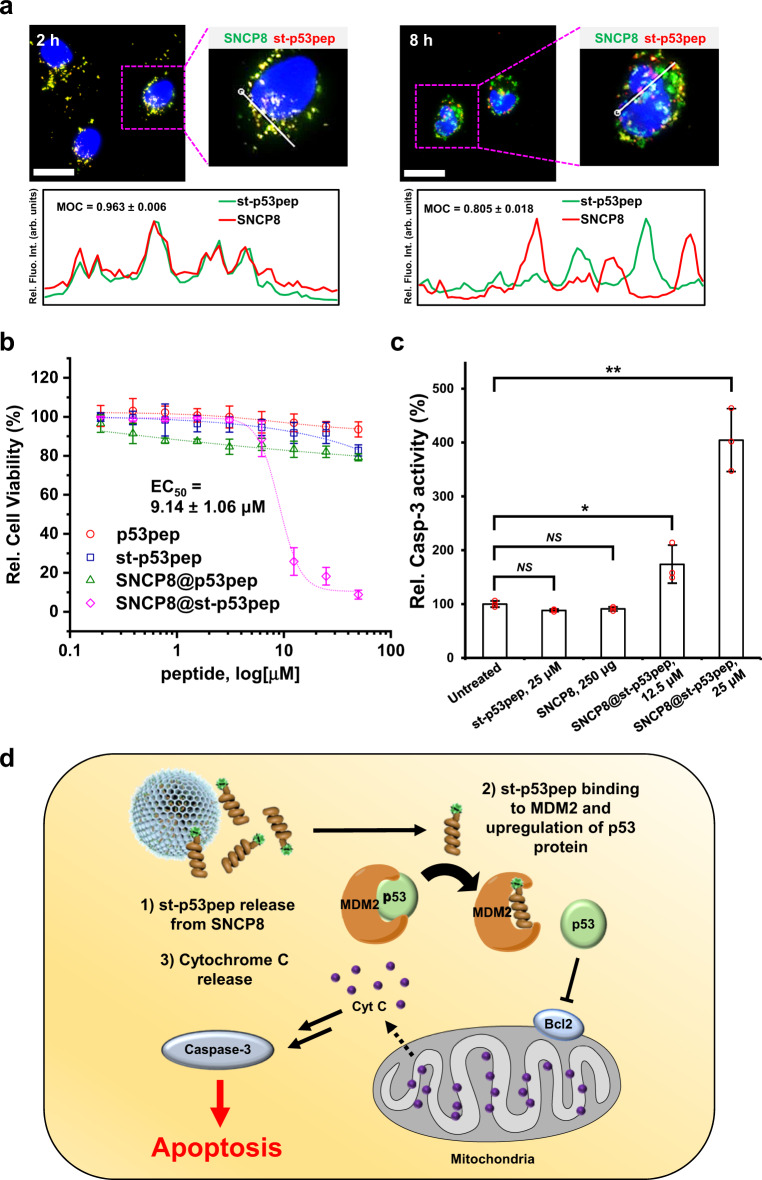
Cell experiments and apoptosis study. **a** Bright field and fluorescence images showing localization of st-p53pep (green, FAM) and SNCP8 (red, TAMRA) in the HepG2 cells 2 and 8 h after treatment of TAMRA-labeled SNCP8@st-p53pep. Relative fluorescence intensities of st-p53pep and SNCP8 were measured along with line scan profile in fixed cells (scale bar = 25 μm). **b** Viability of HepG2 cells treated with various concentrations of SNCP8@st-p53pep and its comparative groups (*n* = 3, biologically independent cells; mean ± SD; red circle: p53pep, blue square: st-p53pep, green triangle: SNCP8@p53pep, magenta diamond: SNCP8@st-p53pep). **c** Relative caspase-3 activities measured from the cells treated with st-p53pep or SNCP8@st-p53pep (*n* = 3 biologically independent samples; mean ± SD; *p < 0.05, **p < 0.01, NS; not significant, unpaired *t* test, The exact *p* values are provided in the Source Data file). **d** Schematic representation of the suggested cell death mechanism via p53-mediated signaling pathway induced by SNCP8@st-p53pep.

Next, we examined efficacy of SNCP as anti-cancer peptide delivery vehicle. Since the apoptotic function of p53 is crucial for tumor suppression, the reconstitution of p53-dependent cell death pathway is an attractive approach to treat cancer^45–47^. Before evaluation of its therapeutic efficacy as a peptide drug, we first measured binding affinity of p53pep and st-p53pep towards its known binding counterpart, MDM2 protein. As expected, the st-p53pep showed higher binding affinity for MDM2 protein (K_d_ = 27.66 ± 1.36 nM) compared to that of p53pep (K_d_ = 80.61 ± 2.33 nM) (Supplementary Fig. 13). To evaluate efficacy of the SNCP8@st-p53pep as a peptide therapy, we first investigated cellular uptake and intracellular distribution of SNCP8@st-p53pep using hepatocellular carcinoma cell line, HepG2. Fluorescence microscopy images showed that green fluorescence of the FAM conjugated to st-p53pep was strongly visualized in the cells treated with SNCP8@st-p53pep (Supplementary Fig. 14). No fluorescence appeared in the cells treated with st-p53pep alone and PBS as a control. To investigate the lysosomal escape of SNCP8@st-p53pep, the lysosomes in HepG2 cells were labeled with lysotracker green after transfection with the SNCP8@st-p53pep. As shown in Supplementary Fig. 15, the yellow fluorescence of SNCP8@st-p53pep was dispersed in the cytoplasm and slightly overlapped the green fluorescence of lysotracker green after incubation for 6 h, indicating that SNCP8@st-p53pep had successfully escaped from lysosome into the cytoplasm. Furthermore, the yellowish signal originated from the co-localization of FAM-st-p53pep (green) and carboxytetramethylrhodamine (TAMRA)-SNCP8 (red) was separated to green and red signals over time in cytoplasm, suggesting that st-p53pep was released from SNCP8 (Fig. 4a)^48^. This result was strongly supported by in vitro data that showed the sustained release of st-p53pep from SNCP8 in buffered solution containing 10% fetal bovine saline (FBS) (Supplementary Fig. 16). Considering that the peptide release from SNCP8 was not readily observed in a buffer solution omitting FBS within the time duration, the release of st-p53pep in early time points might be mainly mediated by surrounding proteins which participate in the hydrophobic interaction with st-p53pep, and subsequent partitioning of st-p53pep, which is in strong accordance with the previous study^49^. Taken together, the data indicated that the present SNCP8@st-p53pep was readily uptaken to the HepG2 cells and the loaded st-p53pep cargo was released from SNCP8 over time inside cells, suggesting its potential as an intracellular delivery vehicle of peptides.

To investigate the cytotoxicity of SNCP8@st-p53pep, we measured relative viability of the cells after incubation with various concentrations of SNCP8@st-p53pep and other comparative groups by using cell counting kit-8 (CCK-8) assay. No notable reduction in cell viability was observed for the SNCP treated groups even at high concentration of 500 μg/mL showing more than 80% survival (Supplementary Fig. 17). In the SNCP8@st-p53pep treated groups, cell viabilities were highly dependent on the concentration of SNCP8@st-p53pep, showing half-maximal effective concentration (EC_50_) of 9.14 ± 1.06 μM, while high cell viabilities observed at corresponding dose of SNCP8@p53pep, p53pep, and st-p53pep groups above 80%. (Fig. 4b). Low cytotoxicity of free peptide-treated groups might be due to lower cellular uptake efficiency of the peptide itself. On the other hand, the delivered p53pep with reversible secondary structure cannot maintain α-helical structure which is required for high binding affinity to MDM2, resulting in little cytotoxicity. These results indicate that the cytotoxicity was successfully induced by the synergistic effects of st-p53pep and SNCP8 rather than SNCP8@p53pep or peptide alone.

To explore cytotoxic mechanism of SNCP8@st-p53pep, we next investigated whether the st-p53pep released from SNCP8 could reactivate the p53-mediated apoptosis pathway. As expected, caspase-3 activity was remarkably increased in the cancer cells treated with SNCP8@st-p53pep in a dose dependent manner, while the cells treated with st-p53pep and SNCP8 alone showed similar caspase-3 activity levels to the untreated control (Fig. 4c.), confirming that the cell death mainly occurred by the p53-mediated apoptosis in the cells treated with SNCP8@st-p53pep^50^. Western blot data showed that protein levels of p53, MDM2, and cytochrome c (CytC) were upregulated, while the level of Bcl2 was notably downregulated in HepG2 cells 48 h after treatment of SNCP8@st-p53pep compared with those in the st-p53pep-treated and untreated cells (Supplementary Fig. 18). The released st-p53pep competitively bind to MDM2 protein, and thus, the degradation of endogenous p53 protein mediated by MDM2 binding and subsequent ubiquitination of p53 was successfully inhibited. Upregulated p53 protein induces the deactivation of Bcl2. Finally, apoptosis is successfully induced through a series of processes including CytC release from mitochondria and the caspase-3 activation. Taken together, these results demonstrated that the apoptosis of the cancer cells was induced by SNCP8@st-p53pep through the p53-dependent pathway (Fig. 4d)^51–53^.

### Therapeutic efficacy of SNCP8@st-p53pep in vivo

**Fig. 5:**
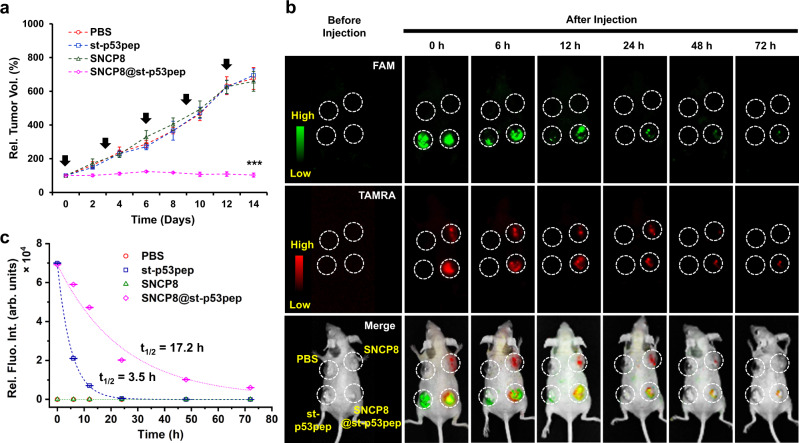
Therapeutic efficacy of SNCP8@st-p53pep investigated in a tumor mouse model and whole body imaging of mice treated with SNCP8@st-p53pep. **a** Time-dependent relative tumor volume of mice after injection with SNCP8@st-p53pep and its comparative groups (*n* = 3, biologically independent animals; 3 days interval; mean ± SD; ***p < 0.001, unpaired *t* test, The exact *p* values are provided in the Source Data file; red circle: PBS, blue square: st-53pep, green triangle: SNCP8, magenta diamond: SNCP8@st-p53pep). Some error bars are too small to be visible. **b** Time-dependent fluorescence images from the whole body of tumor-xenograft mice treated with TAMRA-labeled SNCP8@st-p53pep and its comparative groups (green: st-p53pep, red: SNCP8). **c** Time-dependent relative FAM fluorescence intensity corresponding to st-p53pep (red circle: PBS, blue square: st-53pep, green triangle: SNCP8, magenta diamond: SNCP8@st-p53pep).

To investigate whether SNCP8@st-p53pep could effectively suppress tumor growth in vivo, we evaluated the anti-tumor effect of SNCP8@st-p53pep using human liver cancer (HepG2) xenograft bearing mouse model. Mice were treated with SNCP8@st-p53pep and controls by intratumoral injection every three days and tumor volume changes were monitored for 14 days. The growth of tumor in the SNCP8@st-p53pep treated group was significantly inhibited, while the groups of SNCP8 or st-p53pep alone showed no notable difference in the growth of tumor volume compared to the untreated control (Fig. 5a). These results demonstrated that SNCP8@st-p53pep has an inhibitory effect on tumor growth and SNCP8 could serve as a potent delivery vehicle for the therapeutic anti-tumor peptide in a mouse model. To further examine sustainability of st-p53pep in vivo, we first investigated the proteolytic stability of p53pep inside the SNCP8 in the presence of proteinase K. The semi-quantitative MALDI-TOF showed the rapid degradation of free p53pep within 1 min while the degradation of p53pep inside the SNCP8 is considerably slow, indicating the protective effect of SNCP8 against proteolytic degradation (Supplementary Fig. 19). Taken together, both the characteristics of sustained release and the protective effect of SNCP8 contribute to the sustainability of p53pep in vivo. Next, we monitored distribution of TAMRA-SNCP8 (red), FAM-st-p53pep (green), and TAMRA-SNCP8@FAM-st-p53pep in the tumor-bearing mouse overtime after intratumoral injection. As shown in Fig. 5b and Supplementary Fig. 20, green fluorescence signal in the tumor treated with TAMRA-SNCP8@FAM-st-p53pep slowly diminished during 72 h, while signal in tumor treated with FAM-st-p53pep alone quickly disappeared within 24 h. Gradual attenuation of red fluorescence intensity corresponding to TAMRA-SNCP8 indicated that TAMRA-SNCP8 degrades or dissipate slowly overtime. To compare the lifetime of st-p53pep in tumor more precisely, we calculated the half-life (t_1/2_) of green fluorescence decay for each sample by analyzing region of interest (ROI) on the basis of whole-body fluorescence image (Fig. 5c). The t_1/2_ of st-p53pep inside SNCP8 was 17.2 h, which was about five times longer than that of st-p53pep alone (3.5 h) and maintained weak fluorescent intensity even after 48 h. Collectively, SNCP8 not only served as a nanoreactor to make conformation of p53pep more stable but also served as a peptide carrier to deliver st-p53pep to tumor tissues and cancer cells, sustainably releasing the cargo and effectively inducing p53-mediated apoptosis in vivo.

## Discussion

Multifunctional synthetic nano-chaperone system for peptide was developed, and successfully utilized as a functional peptide delivery system to effectively control tumor growth in vivo. With the elaborated design of aliphatic chain, the hydrophobic interior space inside the SNCP was optimized for stabilizing α-helical structure of the loaded peptide and for facilitating intramolecular hydrogen bonding in peptide backbone. Moreover, the SNCP8 significantly improved the thermal stability of α-helical conformation by reducing the structural degree of freedom of the peptide. The present nano-chaperone system, SNCP8, is operated in three steps; (1) the peptide loading and conformation stabilization under hydrophobic environment in the inner space of porous nanoparticles, (2) the locking of the peptide conformation by click reaction, and (3) the direct injection of the SNCP8@peptide complex for the intracellular delivery to sustainably release cargos and to induce anti-tumor therapeutic effect. The st-p53pep in SNCP8 was effectively released in the cytoplasm of cancer cells, competitively interacted with MDM2, and thus, induced the activation of p53-mediated apoptosis pathway, resulting in cancer cell death. Highly effective anti-tumor therapeutic efficacy was observed in a HepG2 liver cancer xenograft mouse model, showing highly improved half-life of the peptide compared to control groups without SNCP8. Due to significant benefits over conventional methods such as its effective cellular uptake, sustainability, biocompatibility, and flexible administration routes, the SNCP8@peptide complex would provide an attractive option for safe and effective peptide therapy.

Although the peptide stapling strategy itself was demonstrated for therapeutic α-helical peptide candidates in quite a few previous studies, it still requires additional delivery system to ensure efficient cellular uptake and chemical stability against protease-mediated degradation. With the help of SNCP8, st-p53pep was efficiently prepared and exhibited a desired cytotoxic effect in cancer cells by effective intracellular uptake. Our nano-chaperone system, SNCP8, restricts the conformational freedom of the peptide by steric confinement in the porous nanoparticles. In particular, SNCP8 is more effective in improving the proteolytic stability of the encapsulated peptides compared to other strategies studied for stabilizing α-helical conformation where peptides are attached to the surface of nanoparticles.

In conclusion, we successfully developed a synthetic nano-chaperone for peptide based on porous nanoparticles, SNCP, and demonstrated its multiple functions for the stabilization and functional delivery of therapeutic α-helical peptide to control cancer growth. The multifunctionality of SNCP would make it highly attractive for its routine implementation in diverse applications necessitating conformational stability of peptide and its intracellular delivery. We expect that the present SNCP system could be employed as a promising platform for various peptide therapeutics in diverse indications in the future.

## Methods

### Materials

Reagents for SNCP synthesis are as follows: hexadecyltrimethylammonium bromide was purchased from Acros (Belgium). Tetramethyl orthosilicate (TMOS, 98%), (3-aminopropyl)triethoxysilane (APTES, 98%), triethoxy(propyl)silane (97%), triethoxy(octyl)silane (96%), mesitylene (97%), 2-[2-(2-methoxyethoxy)ethoxy] acetic acid (90%), N-(3-Dimethylaminopropyl)-N′-ethylcarbodiimide hydrochloride (EDC, 98%), and N-hydroxysuccinimide (NHS, 98%) were purchased from Sigma (USA). Reagents for p53pep preparation are as follows: Fmoc-protected natural amino acid precursors, activating reagents (HBTU) and solid phase support (Rink amide MBHA resin) were obtained from Beadtech (Korea). (2 s)-N-Fmoc-5-azido-pentanoic acid and Fmoc-D-Lys(Mtt)-OH were purchased from Anaspec (USA). 4-pentynoic acid (98%) and 5(6)-carboxyfluorescein (FAM, 95%) were purchased from Sigma (USA). Other chemical and biochemical reagents are as follows: 5(6)-carboxy-X-rhodamine N-succinimidyl ester (TAMRA, 95%) and Nile red (98%) were purchased from Sigma (USA). Dulbecco’s Modified Eagle’s Medium, fetal bovine serum (FBS) and PBS (pH 7.4) were purchased from Welgene (Korea). Cell Counting Kit-8 (CCK-8) was purchased from Dojindo Molecular Technologies (USA). MDM2_1-118_, a part of the target protein possessing the p53pep binding site, was purchased from SignalChem (Canada). Ac-DEVD-pNA was purchased from Santa Cruz (USA). All of the purchased chemicals were used without further purification.

### Synthesis of p53pep

The p53pep was synthesized on rink amide MBHA resin manually using standard Fmoc SPPS protocols. All of amino acid monomers were coupled manually to resin with the HBTU activation method. 4-pentynoic acid and 5(6)-carboxyfluorescein (FAM) were coupled using a similar method to amino acids. Immediately after coupling of D-Lys, selective deprotection of the 4-methyltrityl (Mtt) group was followed by introduction of 4-pentynoic acid. For peptide deprotection and cleavage, the resin was treated with a cleavage cocktail (TFA: 1,2-ethanedithiol: thioanisole = 95: 2.5: 2.5 v/v) for 3 h and the crude product was collected by precipitation in tert-butyl methyl ether. The p53pep was purified by reverse-phase HPLC (water-acetonitrile with 0.1% TFA v/v) on a C18 column (Vydac). Molecular weight and purity of p53pep were confirmed by MALDI-TOF MS and HPLC (Supplementary Fig. 1c, d). Peptide purity was >95% as determined by analytical HPLC.

### Synthesis of SNCPs

First, amine-functionalized mesoporous silica nanoparticles with organic surfactant in the pores were prepared using the protocol used in a previous report^25^. To introduce triethylene glycol moiety onto the outer surface of the nanoparticles, amine-functionalized mesoporous silica nanoparticles (2 g) were suspended in 30 mL of PBS buffer solution (pH 7.4) with 2-[2-(2-methoxyethoxy)ethoxy]acetic acid (1 mL), EDC (100 mg), and NHS (200 mg). The mixture was stirred at room temperature for overnight. After removing organic surfactant by refluxing in an acid ethanol solution, SNCP0 was collected by centrifugation at 7000 × *g* for 10 min and washed with ethanol several times. To introduce aliphatic chain moieties onto the inner surface of the nanoparticles, SNCP0 (1 g) was suspended in 20 mL of toluene. Then, triethoxy(propyl)silane (1 mL) for SNCP3 or triethoxy(octyl)silane (1 mL) for SNCP8 were added with stirring for overnight. Finally, SNCP3 or SNCP8 were collected by centrifugation at 7000 × *g* for 10 min and washed with ethanol several times. All of SNCPs were dried in a vacuum oven. TAMRA-labeled SNCP8 was prepared using the protocol used in previous studies^25^.

### Preparation of SNCPs@p53pep

The p53pep was dried in glass vial by using a rotational evaporator. A small amount of DMSO (2.5 volume per total volume % for solution) was added to dissolve a thin-film of the dried p53pep, then the DMSO solution color was turned to yellow. Then, the SNCPs aqueous solution (1 mg/mL) was added with stirring for overnight (100 nmole per 1 mg of SNCPs). The orange-colored precipitate was collected by centrifugation at 7000 × *g* for 10 min and rinsed with water.

### Click peptide stapling method

The click peptide stapling was performed through a copper (I) catalyzed alkyne-azide cycloaddition while the peptide is loaded in SNCPs. First, sodium ascorbate (1 mg) was dissolved carefully in 5 mL of CuSO_4_ aqueous solution (1 mM), incubating for 5 minutes at room temperature. SNCPs@p53pep (10 mg) was suspended in 5 mL of water and the prepared aqueous solution of Cu(I) was added with stirring for overnight at 50 °C. Then, SNCPs@st-p53pep were collected by centrifugation at 7000 × *g* for 10 min and washed with water several times to remove CuSO_4_ and sodium ascorbate.

### Characterization

UV-vis absorption spectra were obtained with a UV-2550 (Shimadzu, Japan) and a SynergyMx (Biotek, UK). Fluorescence emission spectra were obtained by using a spectrofluorometer FP-8300 (Jasco, USA). Hydrodynamic sizes and zeta-potentials of the nanoparticles were measured using dynamic light scattering analysis with a Zetasizer NanoS (Malvern instruments, UK)^27^. Reflectance FT-IR spectra were collected using a Hyperion 2000 microscope optically coupled to a Bruker Optics Vertex 70 (Bruker, USA). TEM images were obtained by an energy-filtering transmission electron microscope (EF-TEM) LIBRA 120 (Carl Zeiss, Germany) at 120 kV. Nitrogen adsorption isotherms were measured in a NOVA sorption apparatus. Surface area calculations were carried out using the Brunauer-Emmitt-Teller (BET) method, and pore size distribution was calculated according to the Barrett-Joyner-Halenda (BJH) method^30^.

### Nile red assay

Each SNCP (10 mg) was suspended in 100 μL of Nile red solution (1 mg/mL, in methanol) with stirring for 1 h. These complexes were collected by centrifugation at 7000 × *g* for 10 min and were washed alternately with methanol and water several times to remove adsorbed Nile red on the nanoparticle surface. Fluorescence emission spectra of the adsorbed Nile red were measured (λ_ex_ = 510 nm) and relative emission intensities were compared for each complex solution.

### CD spectroscopy and analysis

CD spectra were measured using a J-810 spectropolarimeter (JASCO, Japan). Spectra were recorded from 250 nm to 190 nm wavelength using a 1 cm path-length cuvette. Scans were repeated five times and averaged. Molar ellipticity was calculated per amino acid residue. The concentrations of SNCP and p53pep were 0.2 mg/mL and 20 μM, respectively. All sample suspensions were incubated at least for 2 days at room temperature before CD measurement. To perform temperature-dependent CD experiments, the spectra were recorded at 10 °C interval over a temperature range of 25 to 75 °C, with 30 min incubation time to reach equilibrium at each temperature. The α-helicity of peptides was calculated by using the CONTIN-LL algorithms provided from the DICHROWEB server^37^.

### Binding affinity analysis

Each of p53pep and st-p53pep (50 nM) were incubated with MDM2_1-118_ with various concentrations in binding assay buffer (PBS pH 7.4 with 0.01% Tween20, 10 mM DTT, 2% DMSO) at room temperature. Binding affinity was measured by fluorescence polarization on a Biotek Synergy Neo2 multi-mode microplate reader. K_d_ values were determined by nonlinear regression analysis.

### In vitro release experiment

SNCP8@st-p53pep (5 mg) was suspended in 5 mL of PBS buffer or PBS buffer with 10% FBS. And, these solutions kept with stirring at room temperature (*n* = 3, biologically independent experiments). At various intervals over a total of 7 days, the supernatant was collected by using centrifugation at 7000 × *g* and 4 °C for 10 min. The concentration of the released st-p53pep in the supernatant was calculated by measuring fluorescence intensity of FAM attached to st-p53pep (λ_ex_ = 480 nm, λ_em_ = 520 nm).

### Cell experiments

Human liver cancer cell line HepG2 were grown in DMEM containing 10% FBS and 1% penicillin/streptomycin at 37 °C in a humidified atmosphere of 5% CO_2_. HepG2 cells (1.2 × 10^5^ cells/well) were seeded in a 4-well glass plate for 24 h. Then, SNCPs@st-p53pep (0.02 mg/mL) and st-p53pep alone (2 μM) were added to each well in a serum-free medium for 4 h. After incubation, the cells were carefully rinsed with PBS, and the medium was replaced with a serum-containing fresh medium. After the nucleus was stained with Hoechst 33342 staining kit by manufacturer’s protocol, bright field and fluorescence images were obtained by using a DeltaVision Elite Microscopy System (GE Healthcare, USA). To observe the distribution of SNCP8 and st-p53pep in cells, TAMRA-labeled SNCP8@FAM-labeled st-p53pep (0.25 mg/mL) was treated in the same manner instead of SNCP8@st-p53pep. And then, the Mander’s overlap coefficient (MOC) was calculated in the Image J program (ver. 1.52a) via the JACoP plugin to identify co-localization between fluorescence signals over time. To measure cellular toxicity, HepG2 cells (1 × 10^4^ cells/well) were cultured in a 96-well plate in triplicate for 24 h, following incubation with various concentrations of SNCPs and SNCPs@st-p53pep with complete medium. After incubation, the cells were carefully rinsed with PBS. The cell viability was determined using cell counting kit-8 (CCK-8) assay by monitoring absorbance at 450 and 670 nm wavelength measured using a microplate reader (Molecular Devices, Inc., USA). The EC_50_ values of SNCPs@st-p53pep were determined by the logistic curve-fitting method with Origin (OriginLab, USA).

### Apoptosis study

To conduct the caspase-3 activity assay, HepG2 cells were treated with SNCP8, SNCP8@st-p53pep, and PBS as a control for 4 h, and further incubated with fresh media for 20 h. The cells were washed and treated with lysis buffer (Promega, USA), followed by centrifugation at 2000 × *g* and 4 °C for 10 min. The collected lysate was incubated with a caspase-3 substrate (Ac-DEVD-pNA, 100 μM) in a humidified incubator at 37 °C for 2 h. The fluorescence intensity was then measured (λ_ex_ = 400 nm, λ_em_ = 505 nm).

### Western blot

Cells were lysed with Pro-PREP (IntronBio, Korea) according to the manufacturer’s protocol. The samples were centrifuged at 2000 × *g* and 4 °C for 15 min. The supernatant was collected to a 1.5 mL ep-tube and stored at −80 °C. After protein quantification using Pierce^TM^ BCA assay kit (Thermo Fisher Scientific, USA), the same amount of protein was diluted in water containing 5×loading buffer (Biosesang, Korea) and loaded on SDS-PAGE gel (Bio-rad, USA). After electrophoresis in Tris-Glycine SDS buffer at 80 V for 120 min, proteins in a gel were transferred to a nitrocellulose membrane (0.45 μm, Bio-rad, USA) in 1× Tris-Glycine Native Buffer (GenDEPOT, Korea) with 10% methanol at 90 V, and 4 °C for 90 min under stirring condition. The membrane was blocked with 5% milk to inhibit non-specific binding and immersed overnight at 4 °C in the presence of primary antibodies; GAPDH (1:2000, Abcam, USA), p53 (1:1000, Cell signaling, USA), MDM2 (1:1000, Cell signaling, USA), Bcl2 (1:1000, Cell signaling, USA) and CytC (1:1000, Cell signaling, USA). Anti-rabbit goat pAb, horseradish peroxidase (HRP)-linked antibody (1:5000, Abcam, USA) was used as a secondary antibody and the membrane was incubated for 1 h. After the reaction with the detection agent West Pico PLUS (Thermo Fisher Scientific, USA), bands of protein were monitored by a ImageQuant^TM^ LAS 4000 mini (GE Healthcare, USA). All samples were derived from the same experiment and processed in parallel.

### In vivo experiments

All of animal experiments were conducted in accordance with the Institutional Animal Care and Use Committees (IACUC) of Seoul National University. 5-week old Balb/c male nude mice were purchased (ORIENT BIO, Korea). Mice were housed in an environmentally controlled room (23 °C, with 55 ± 5% humidity and 12 h / 12 h light–dark cycle). To prepare tumor-bearing mice, a suspension of the HepG2 cells (6 × 10^6^ cells) in 100 µL of sterilized PBS was subcutaneously injected (*n* = 3, biologically independent animals). Tumor-bearing mice were reared until the tumor volumes reach ~50 mm^3^ and used for experiments. To investigate tumor growth inhibition efficiency, SNCP8@st-p53pep, SNCP8, and st-p53pep alone in 100 μL of PBS solution (50 mg SNCP8/kg, 5 μmole st-p53pep/kg) were directly injected into the tumor. The same volume of PBS solution was also directly treated into the tumor as a control. Intratumoral injection was performed at 3 day intervals. The changes in tumor volume and body weight were monitored over 14 days. Tumor volumes were calculated by using the equation of 1/2 × length × (width)^2^, where the length and width are the longest and shortest diameters (mm) of the tumor, respectively. The relative tumor volume was derived from comparing it with the initial tumor volume. To observe sustainability and tumor exposure of st-p53pep in vivo, we used nude mice with HeLa and HepG2 cell xenografts prepared in the same manner as the tumor growth inhibition experiment. TAMRA-labeled SNCP8@ FAM-labeled st-p53pep was treated in the same manner as the tumor growth inhibition experiment instead of SNCP8@st-p53pep. Bright field and fluorescence images from mice were obtained overtime for 3 days by using fluorescence in vivo imaging system, FoBI (Neoscience, Korea). The relative intensity of FAM fluorescence attached to st-p53pep was calculated by using the region of interest (ROI) analysis. The half-life of FAM fluorescence decay was determined by exponential curve fitting method with Origin 2017 (OriginLab, USA).

### Statistical analysis and reproducibility

All images were obtained from at least three independent experiments with similar results. Also, all data were shown mean corrected values ± SD of at least three independent experiments unless otherwise stated. Graphing of the data was conducted using Microsoft Office Excel and Power Point 2016. Significant differences were determined based on the student’s *t*-test for which the differences were considered significant. Asterisks in figure legends indicate *P*-value thresholds of < 0.05 (*), < 0.01 (**), or < 0.001 (***) from unpaired *t* test. And, *NS* in figure legends means no significance, *p*-value thresholds of > 0.05. Statistical analyses were conducted using GraphPad Prism 7 (GraphPad Inc., USA).

### Reporting summary

Further information on research design is available in the Nature Research Reporting Summary linked to this article.

## Supplementary information

Supplementary Data & Information

Reporting Summary

## Data Availability

The data generated or analysed in this study are provided in the Supplementary Information. Data is available from the corresponding author upon request. Source data are provided with this paper.

## Source data

Source Data
