# Supplementary material for: Multifunctional synthetic nano-chaperone for peptide folding and intracellular delivery
Source: Nat Commun. 2022 Aug 5;13:4568. doi: 10.1038/s41467-022-32268-2 (PMC9356039; doi:10.1038/s41467-022-32268-2)
Supplement: Supplementary file 1 — Supplementary Data & Information [file 41467_2022_32268_MOESM1_ESM.pdf]

Supplementary Information

Multifunctional Synthetic Nano-Chaperone for Peptide Folding and Intracellular Delivery

Il-Soo Park[1†], Seongchan Kim[2†], Yeajee Yim[1], Ginam Park[1], Jinahn Choi[1], Cheolhee Won[3] and Dal-Hee Min[1, 3, *]

[1] Department of Chemistry, Seoul National University, Seoul 08826, Republic of Korea

[2] Biomaterials Research Center, Biomedical Research Division, Korea Institute of Science and Technology (KIST), Seoul 02792, Republic of Korea

[3] Institute of Biotherapeutics Convergence Technology, Lemonex Inc., Seoul 06683, Republic of Korea

† These authors contributed equally.

*Correspondence: dalheemin@snu.ac.kr, +82-2-880-4338 (phone), +82-2-889-1568 (fax)

a

Sequence : **FAM-GGQSQQTFK*NLWRLLK**QN-NH_2**
(**K* :** d-Lysine conjugated pentynoic acid,
K :** 2-amino-5-azido-pntanoic acid)

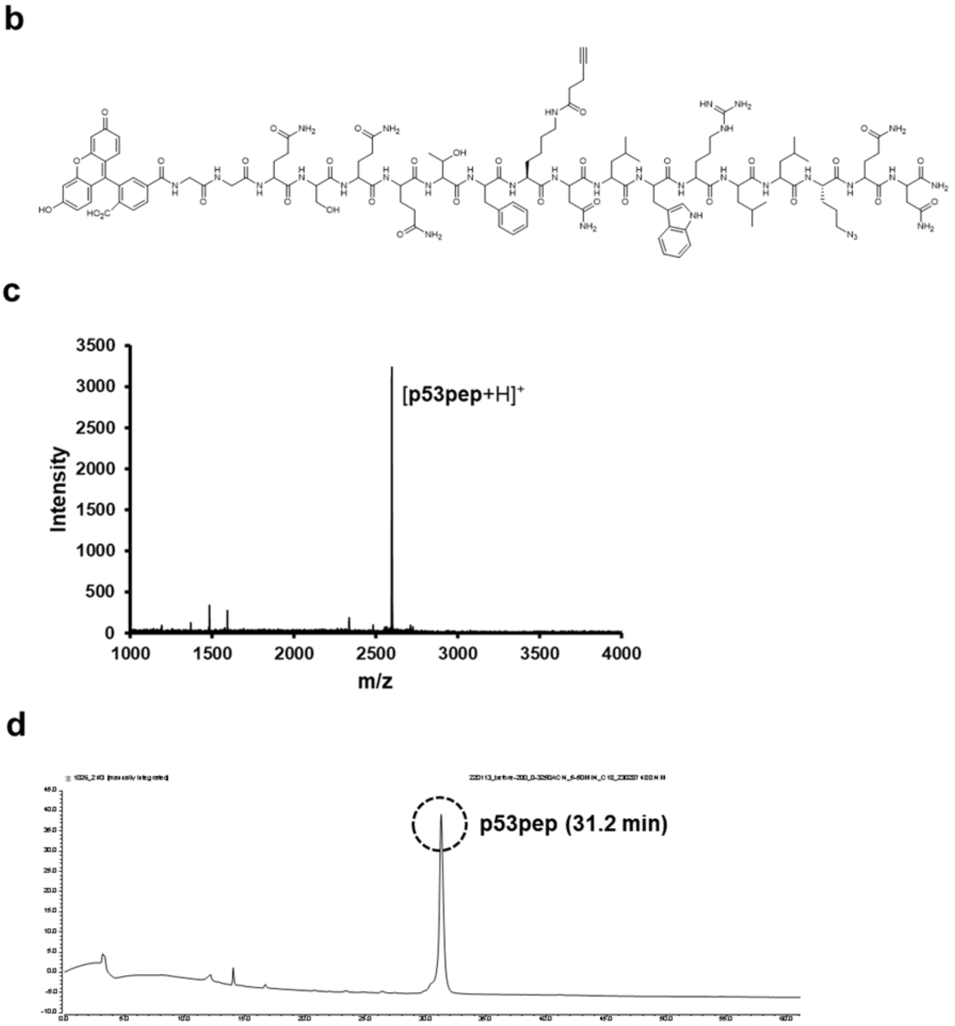

Supplementary Figure 1. Preparation of peptide. (a) Peptide sequence and (b) chemical structure of p53pep. K* (d-Lysine conjugated pentynoic acid) and K** (2-amino-5-azido-pntanoic acid) are analogs of lysine to introduce a reaction site for click stapling. (c) The matrix-assisted laser desorption ionization time-of-flight (MALDI-TOF) mass spectrum of p53pep (C120H163N33O33 – $[p53pep+H]^+$) cal. 2596.78, found 2597.92. (d) High performance liquid chromatography (HPLC) chromatogram of p53pep observed at 230 nm UV absorption. HPLC condition: C18 column (Vydac 218TP, 5μm, C18, 250 × 4.6 mm), room temperature (25°C), flow rate 1 mL/min; ACN/Water 0% for 5 min and gradient mobile phase (32~50%) for 50 min (5~55 min); Retention time: 31.2 min.

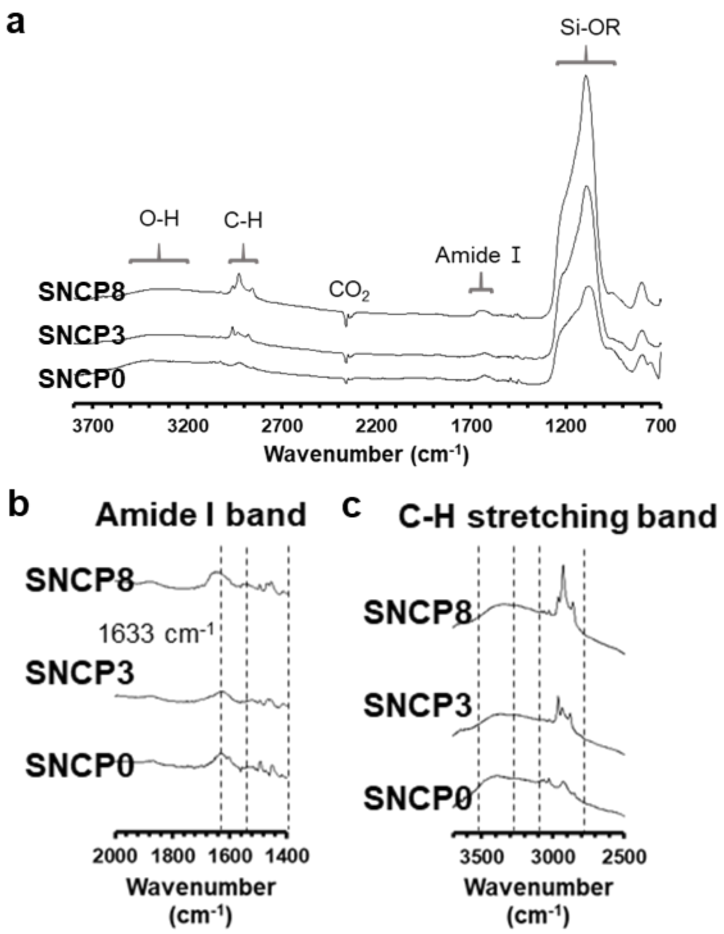

SNCP0	SNCP3	SNCP8
1417	1409	1410
1450	1456	1454
1471	1466	1470
1491	1493	1493

SNCP0	SNCP3	SNCP8
2852	2877	2858
2927	2933	2926
	2962	2960
3026	3024	3025

Supplementary Figure 2. Fourier-transform infrared (FT-IR) spectra of SNCPs. (a) Spectra in a broad wavelength, **(b)** Amide I band from covalent attachment site of TEG on the outer surface, **(c)** C-H stretching band from aliphatic chain moieties on the inner surface.

	Mean pore size (nm)	BET surface area (m^2/g)	Pore volume (mL/g)	p53pep loading capacity (w/w%)
**SNCP0**	12.3±0.05	327±1.7	1.31±0.04	9.96±0.4
**SNCP3**	9.5±0.03	314±4.3	1.14±0.05	12.2±0.3
**SNCP8**	9.3±0.39	297±2.9	1.03±0.02	12.6±0.3

Supplementary Figure 3. Nitrogen adsorption/desorption analysis for SNCPs and p53pep loading capacities of SNCPs. Through nitrogen adsorption experiments, mean pore sizes, surface areas, and pore volumes were collected (N = 3; mean ± SD). The pore size distribution and the surface area were calculated using Barrett-Joyner-Halenda (BJH) method and the Brunauer-Emmtt-Teller (BET) method, respectively. The p53pep loading capacity of SNCPs was calculated by using UV/vis spectroscopy and standard curve of absorbance of FAM labeled on p53pep.

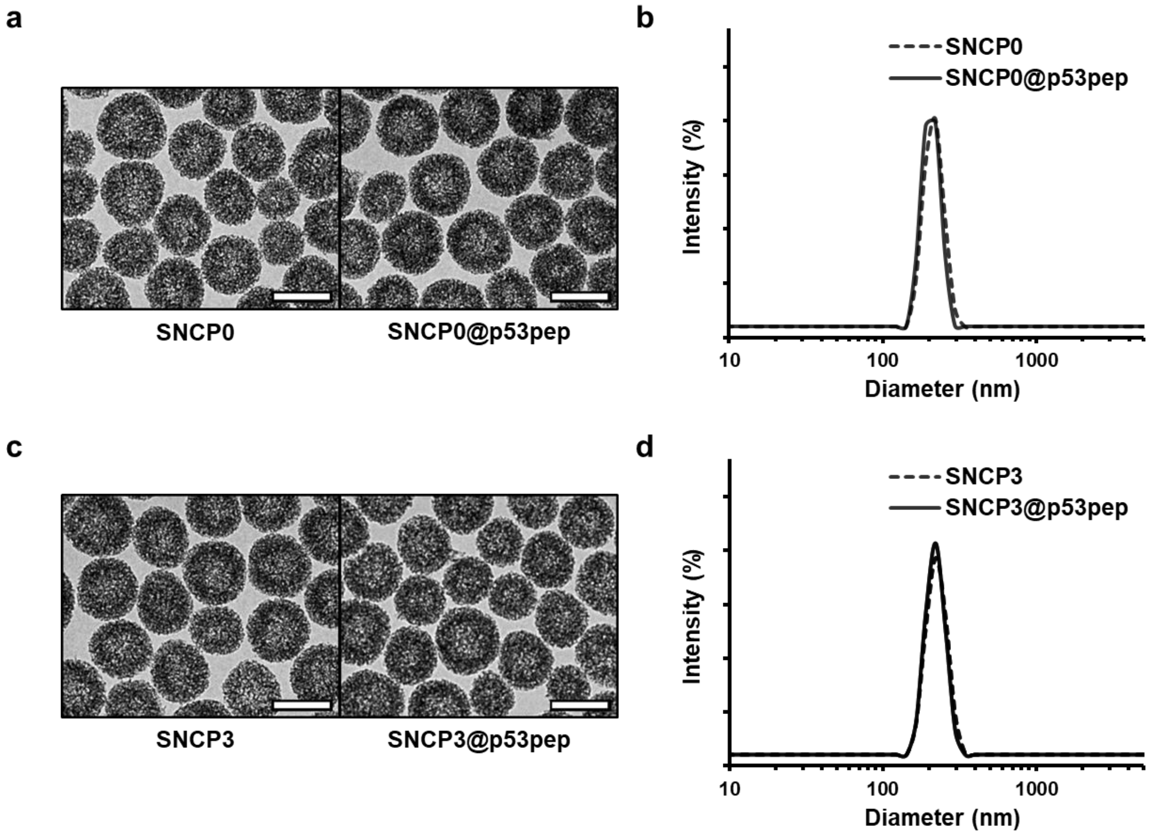

e

	Polydispersity index (PDI)	Z-average (nm)	Size from TEM (nm)
SNCP0	0.076 ± 0.007	181.9 ± 5	183.1 ± 11.8
SNCP0@p53pep	0.095 ± 0.037	181.6 ± 2.7	183.9 ± 9.6
SNCP3	0.091 ± 0.021	184.4 ± 3.3	183.9 ± 10.8
SNCP3@p53pep	0.084 ± 0.028	183.6 ± 2.5	183.5 ± 10.4
SNCP8	0.075 ± 0.007	183.7 ± 2.4	184.4 ± 11.3
SNCP8@p53pep	0.080 ± 0.017	182.7 ± 2.1	184.0 ± 11.5

Supplementary Figure 4. Size distribution of SNCP0 and SNCP3. (a) Transmission electron microscopy (TEM) image and (b) dynamic light scattering (DLS) data of SNCP0 and SNCP0@p53pep. (c) TEM image and (d) DLS data of SNCP3 and SNCP3@p53pep. (e) Polydispersity index (PDI) and Z-average values of SNCPs and SNCPs@p53pep obtained through DLS analysis (N = 10; mean ± SD). Mean size of SNCPs and SNCPs@p53pep calculated from TEM image (analyzed by using image J software; N = 20; mean ± SD).

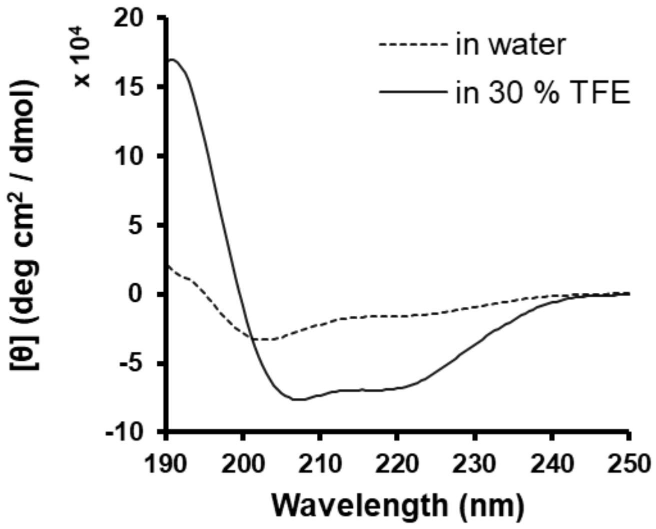

Supplementary Figure 5. Circular dichroism (CD) spectra of p53pep in water and in 30 % 2, 2, 2-trifluoroethanol (TFE) aqueous solution. The CD measurement was carried out at the concentration of 40 μM. The CD spectrum of p53pep in water showed the negative peak at 202 nm, demonstrating random-coil structure. Meanwhile, the CD spectrum of p53pep in 30 % TFE showed two negative peaks at 208 nm and 222 nm, indicating typical α-helical structure.

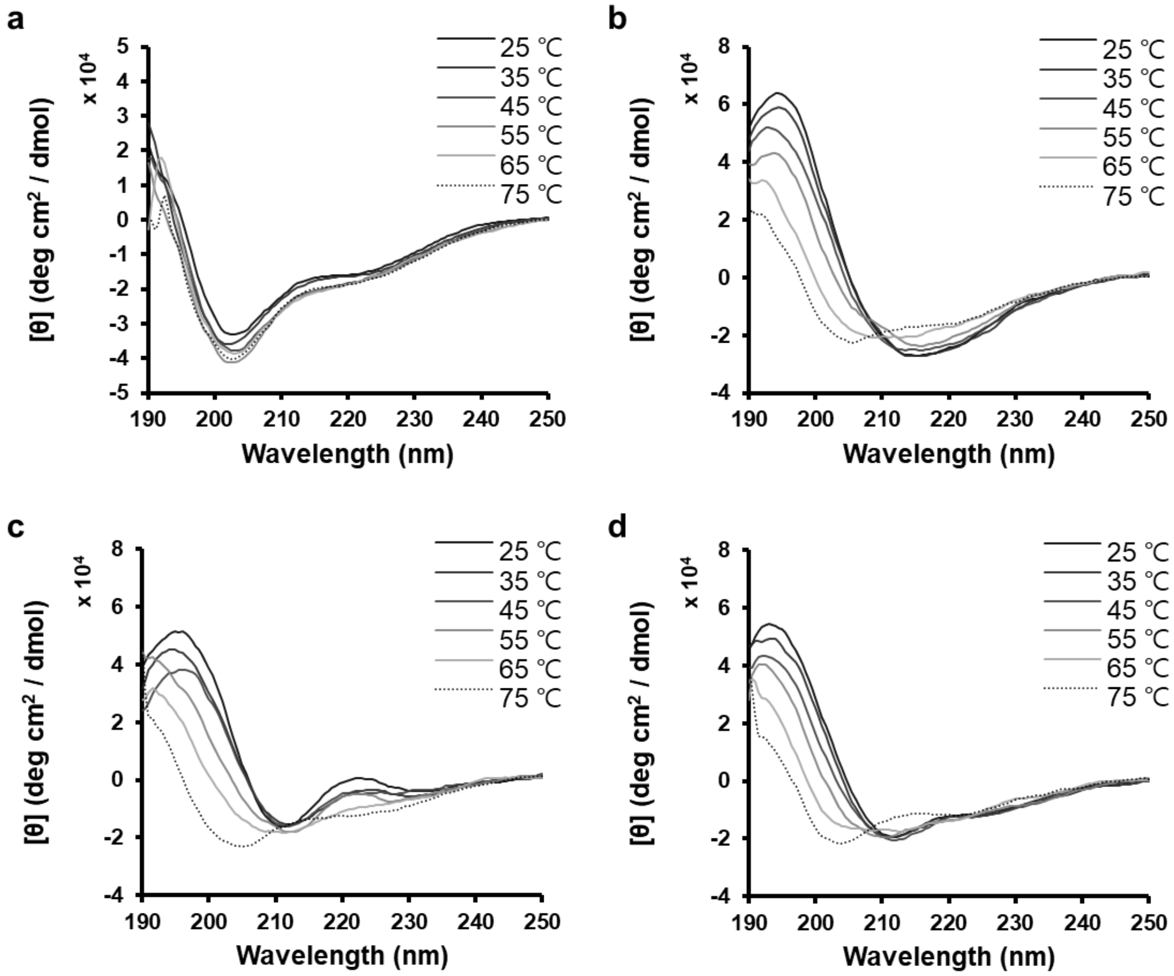

Supplementary Figure 6. The behavior of p53pep according to temperature change. Temperature-dependent circular dichroism (CD) spectra of **(a)** p53pep itself, **(b)** SNCP0@p53pep, **(c)** SNCP3@p53pep, and **(d)** SNCP8@p53pep. The spectra were scanned at 10 °C intervals over a temperature range of 25 to 75°C. An incubation time of 30 minutes was given for each temperature to reach equilibrium.

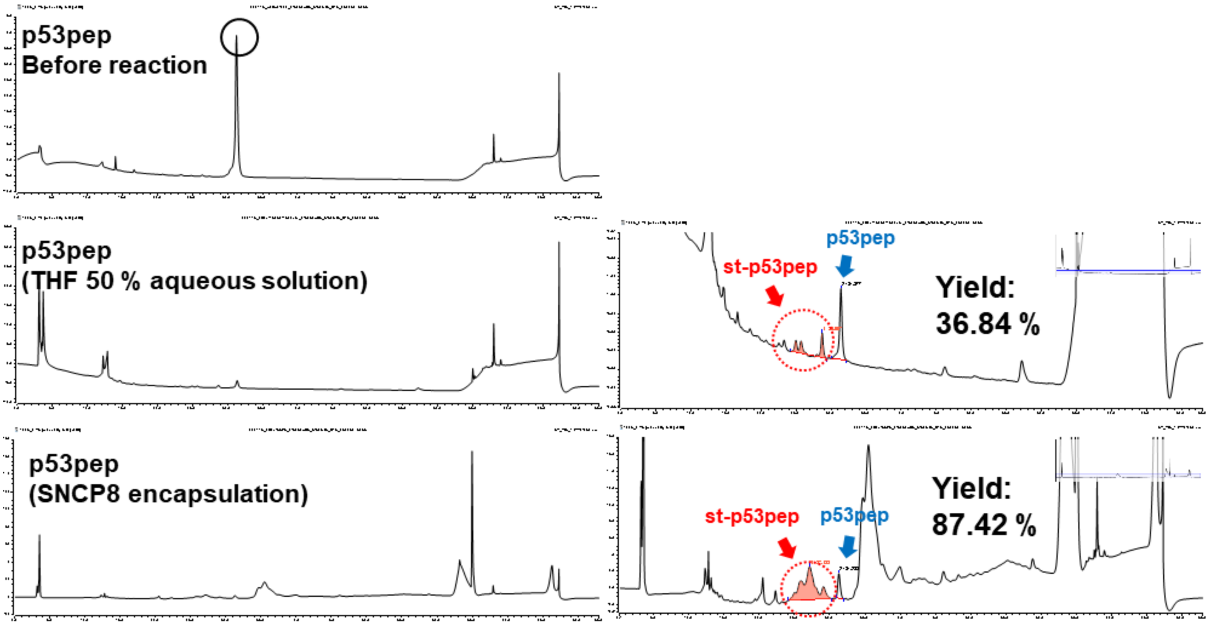

Supplementary Figure 7. The reaction yields according to the click reaction of p53pep. High performance liquid chromatography (HPLC) chromatograms of p53pep. Characteristic spectra of p53pep, p53pep in THF 50% aqueous solution, and p53pep with SNCP encapsulation. HPLC condition: C18 column (Vydac 218TP, 5μm, C18, 250 × 4.6 mm), room temperature (25°C), flow rate 1 mL/min; ACN/Water 0% for 5 min and gradient mobile phase (32~50%) for 50 min (5~55 min); Retention time: 31.2 min for p53pep, 24~30.5 min for st-p53pep, and 33.5~38 min for BSA (as additive for peptide extraction).

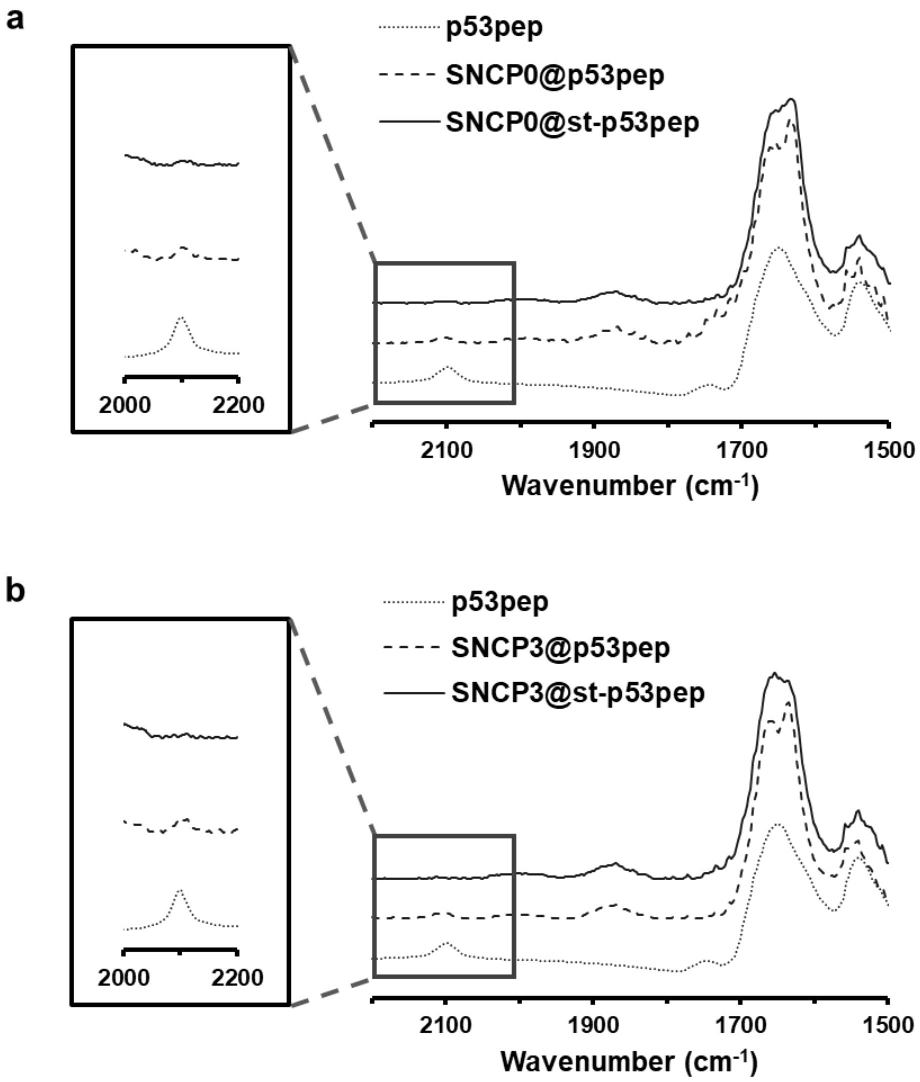

Supplementary Figure 8. Changes in azide functional group according to the click reaction of p53pep. Fourier-transform infrared (FT-IR) spectra of st-p53pep in **(a)** SNCP0 and **(b)** SNCP3. For each of FT-IR spectrum, the region (between 2000 and 2200 cm^{-1}) near azide stretching band was shown in enlarged image.

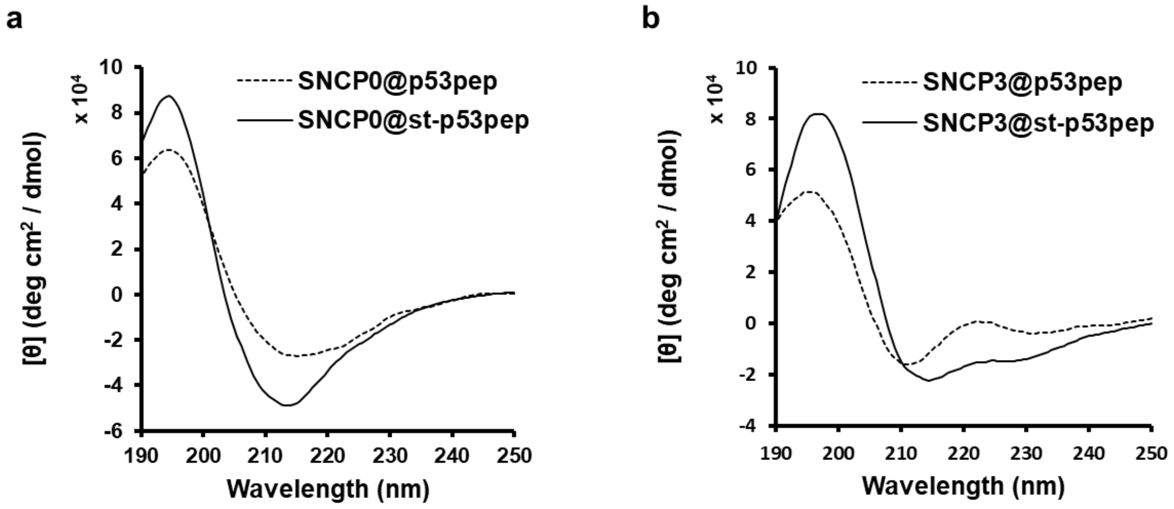

Supplementary Figure 9. The behavior of p53pep in SNCP0 and SNCP3 under peptide stapling. Circular dichroism (CD) spectra of st-p53pep in **(a)** SNCP0 and **(b)** SNCP3. In both graphs, molar ellipticity values were observed to be enhanced.

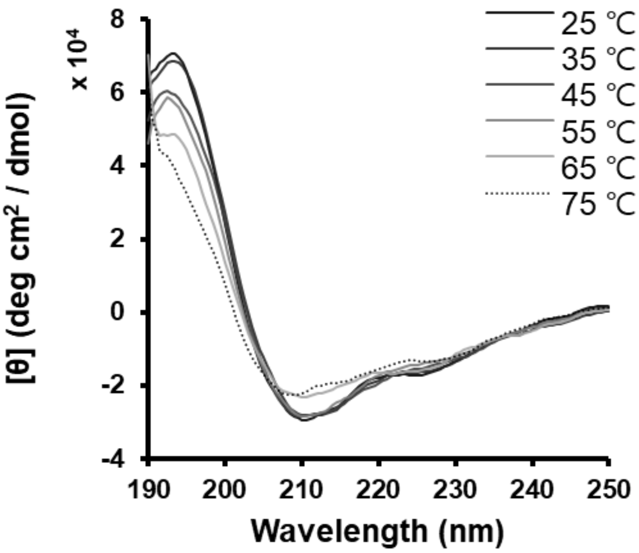

Supplementary Figure 10. The behavior changes according to temperature of st-p53pep in SNCP8. Temperature-dependent circular dichroism (CD) spectra of SNCP8@st-p53pep. The spectra were scanned at 10 °C intervals over a temperature range of 25 to 75 °C. An incubation time of 30 minutes was given for each temperature to reach equilibrium.

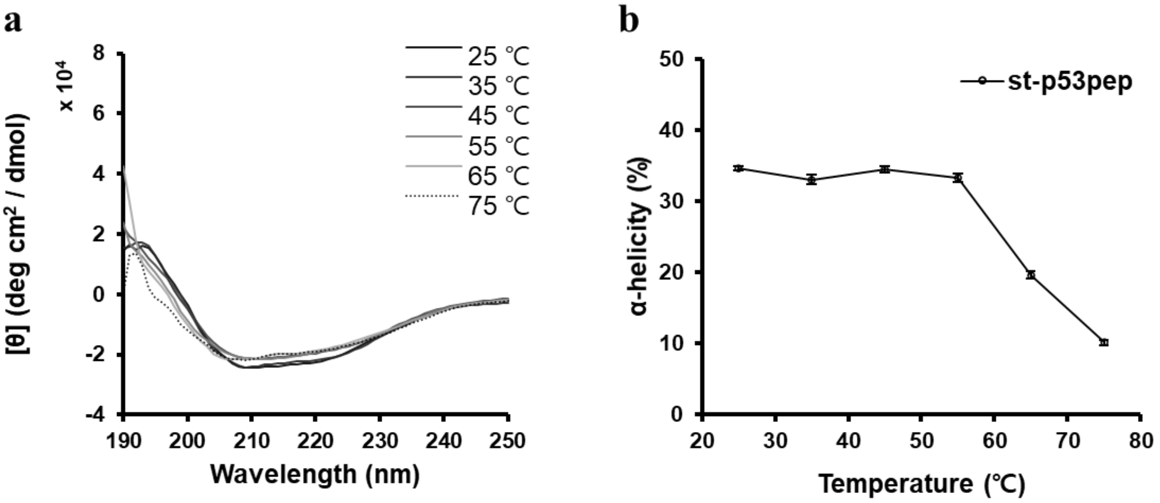

Supplementary Figure 11. The behavior of st-p53pep according to temperature change. Temperature-dependent **(a)** circular dichroism (CD) spectra and **(b)** α-helicity of st-p53pep itself (N = 3; mean ± SD).

a

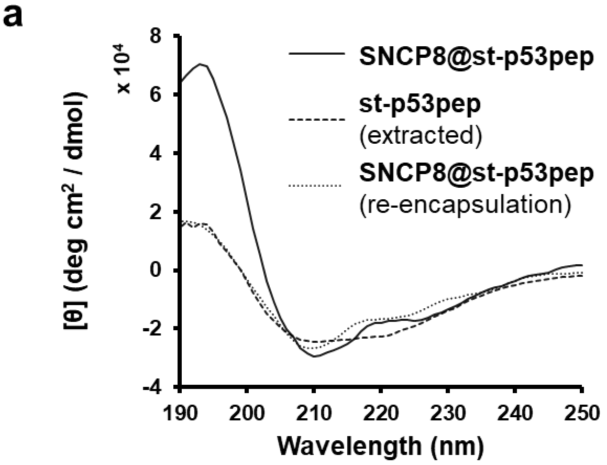

b

	α-helicity %
**SNCP8@st-p53pep**	84.9
**st-p53pep** (extracted)	34.6
**SNCP8@st-p53pep** (re-encapsulation)	73.4

Supplementary Figure 12. The α-helicity changes under various conditions for st-p53pep. (a) Circular dichroism (CD) spectra and **(b)** α-helicity of st-p53peps in various conditions: st-p53pep with SNCP encapsulation, extracted st-p53pep after SNCP encapsulation, and st-p53p with SNCP8 re-encapsulation.

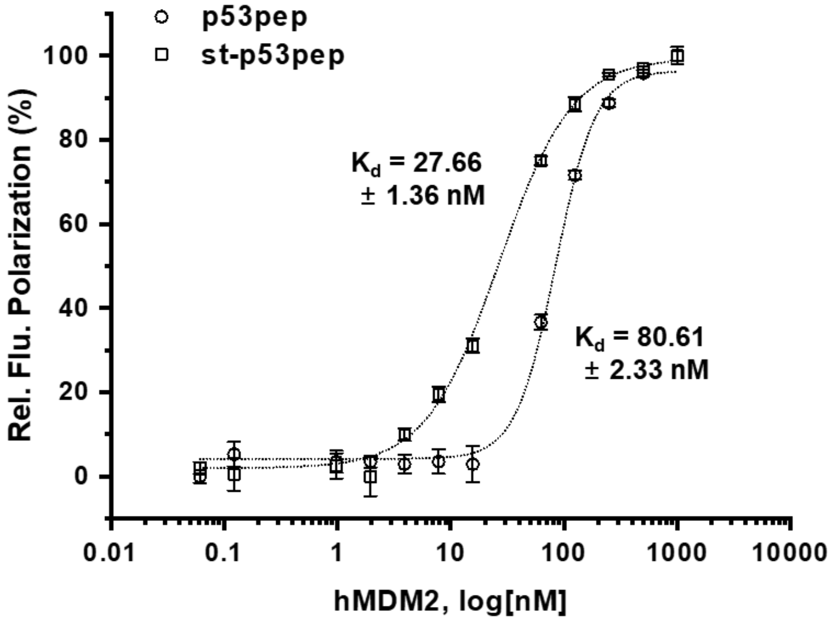

Supplementary Figure 13. The binding affinities of p53pep and st-p53pep to hMDM2 protein. Kd value of p53pep (circle, 80.61 ± 2.33 nM) was higher than that of st-p53pep (square, 27.66 ± 1.36 nM) (N = 3, biologically independent samples; mean ± SD).

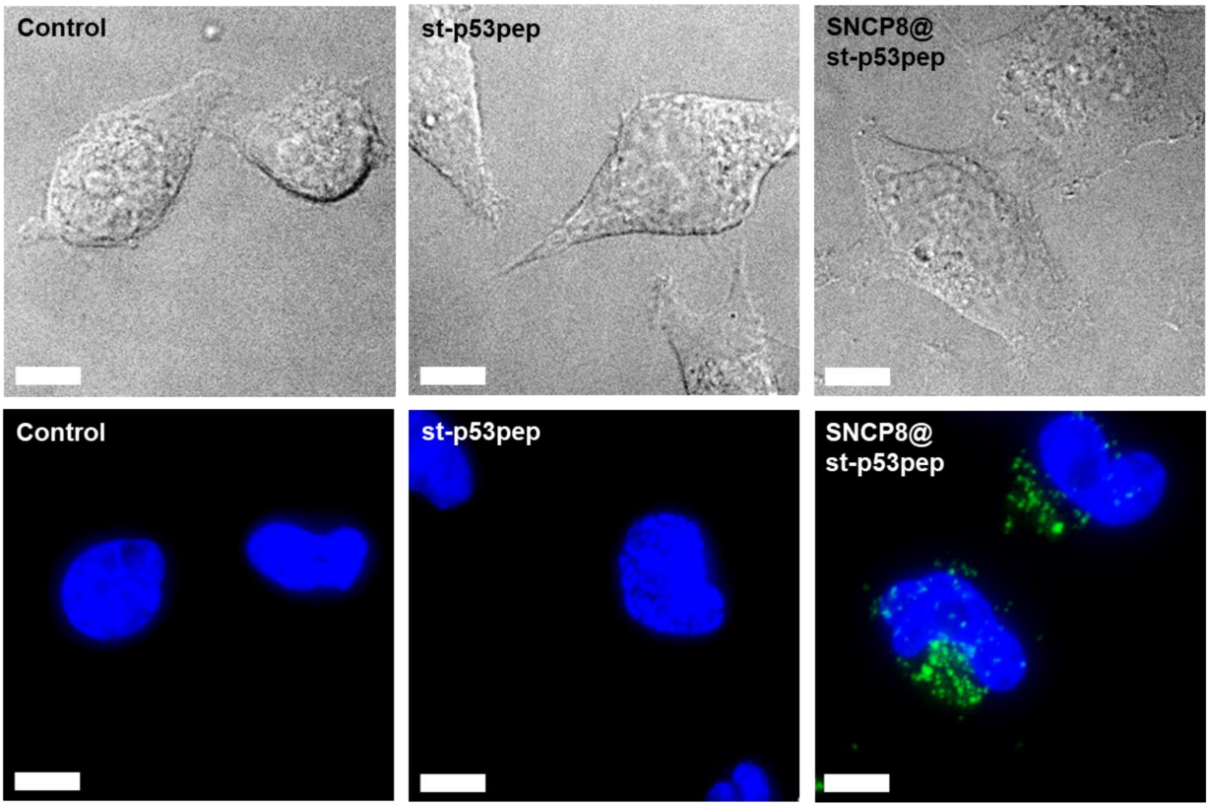

Supplementary Figure 14. Intracellular uptake of SNCP8@st-p53pep. Bright field and fluorescence images demonstrating uptake of st-p53pep itself and SNCP8@st-p53pep into HepG2 Cells (scale bar = 10 μm).

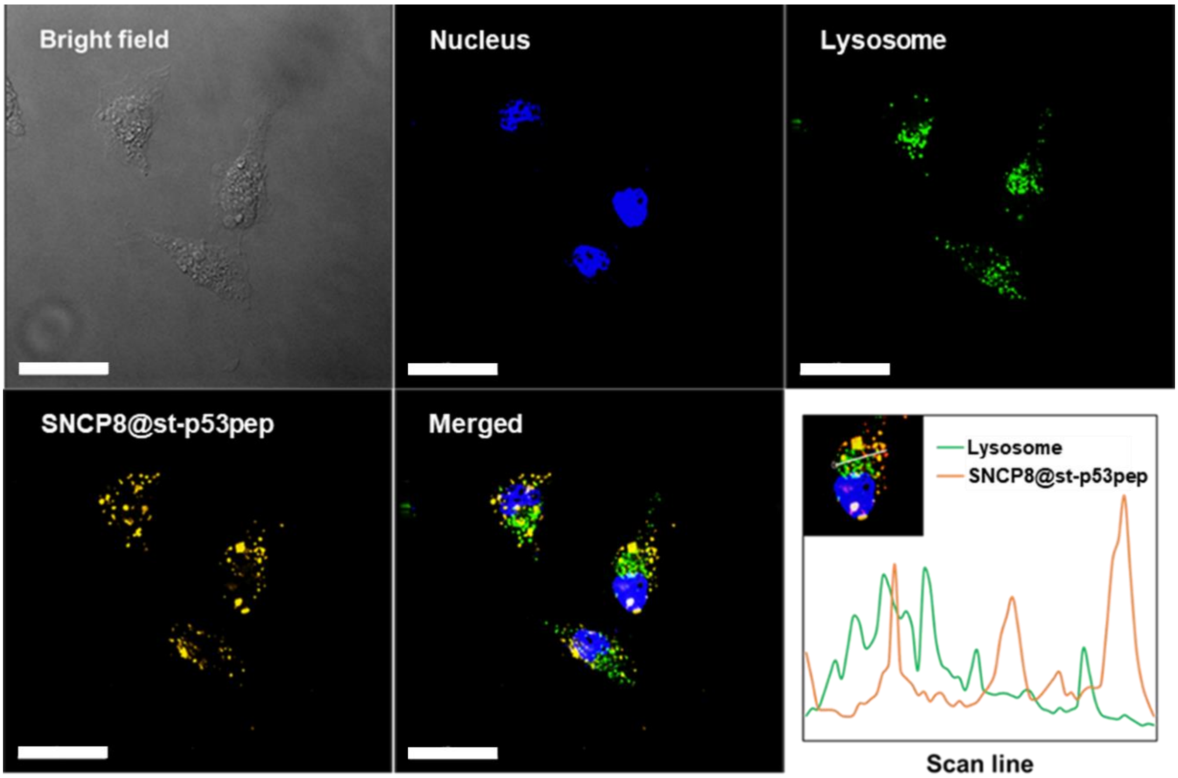

Supplementary Figure 15. Endosomal escape of SNCP8@st-p53pep. Lysosomal escape and distribution of SNCP8@st-p53pep (Green: Lysotracker, Yellow: SNCP8@st-p53pep) (scale bar = 15 μm).

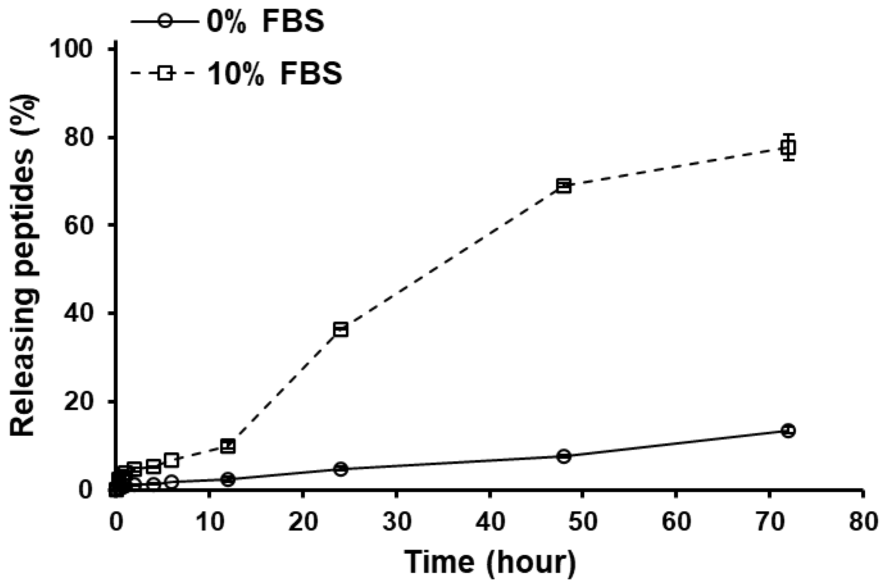

Supplementary Figure 16. Cumulative release study *in vitro*. *In vitro* releasing experiment for SNCP8@st-p53pep (100 μM) in PBS buffer with 10 % FBS (circle: 0% FBS, square: 10% FBS). SNCP8@st-p53pep was dissolved in PBS buffer with 10 % FBS incubating with stirring at 37 °C (N = 3, biologically independent experiments; mean ± SD). Some error bars are too small to be visible. At various intervals over 72 h, the supernatant was collected by using centrifugation. Relative fluorescence intensity of FAM labeled st-p53pep was detected from the supernatant (λ_{ex} = 480 nm, λ_{em} = 520 nm).

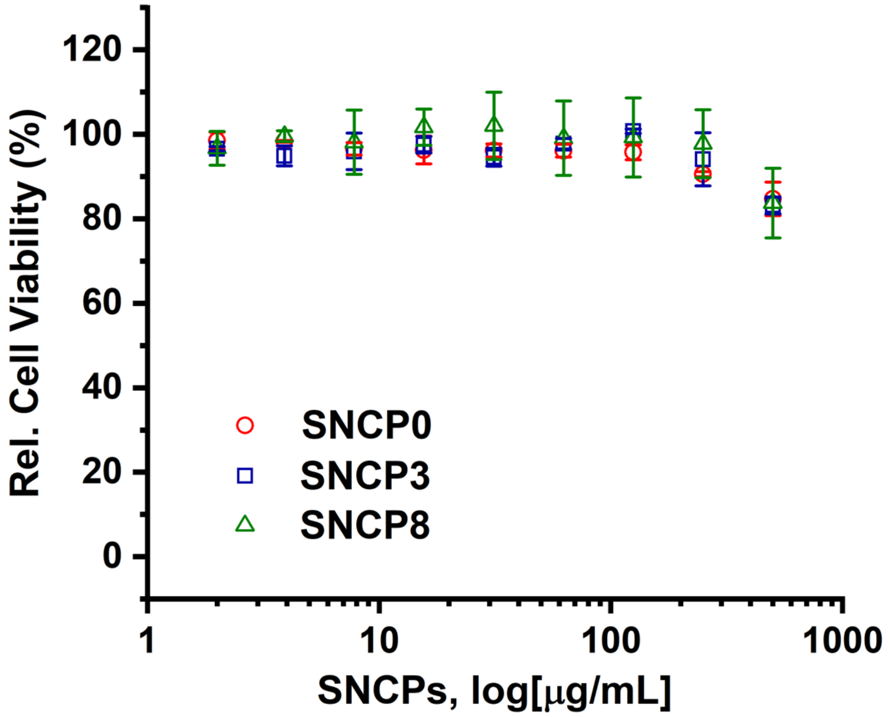

Supplementary Figure 17. Viabilities of SNCPs in cells. Cell cytotoxicity in HepG2 cells with various concentrations of SNCPs (N = 3, biologically independent cells; mean ± SD; red circle: SNCP0, blue square: SNCP3, green triangle: SNCP8). Some error bars are too small to be visible.

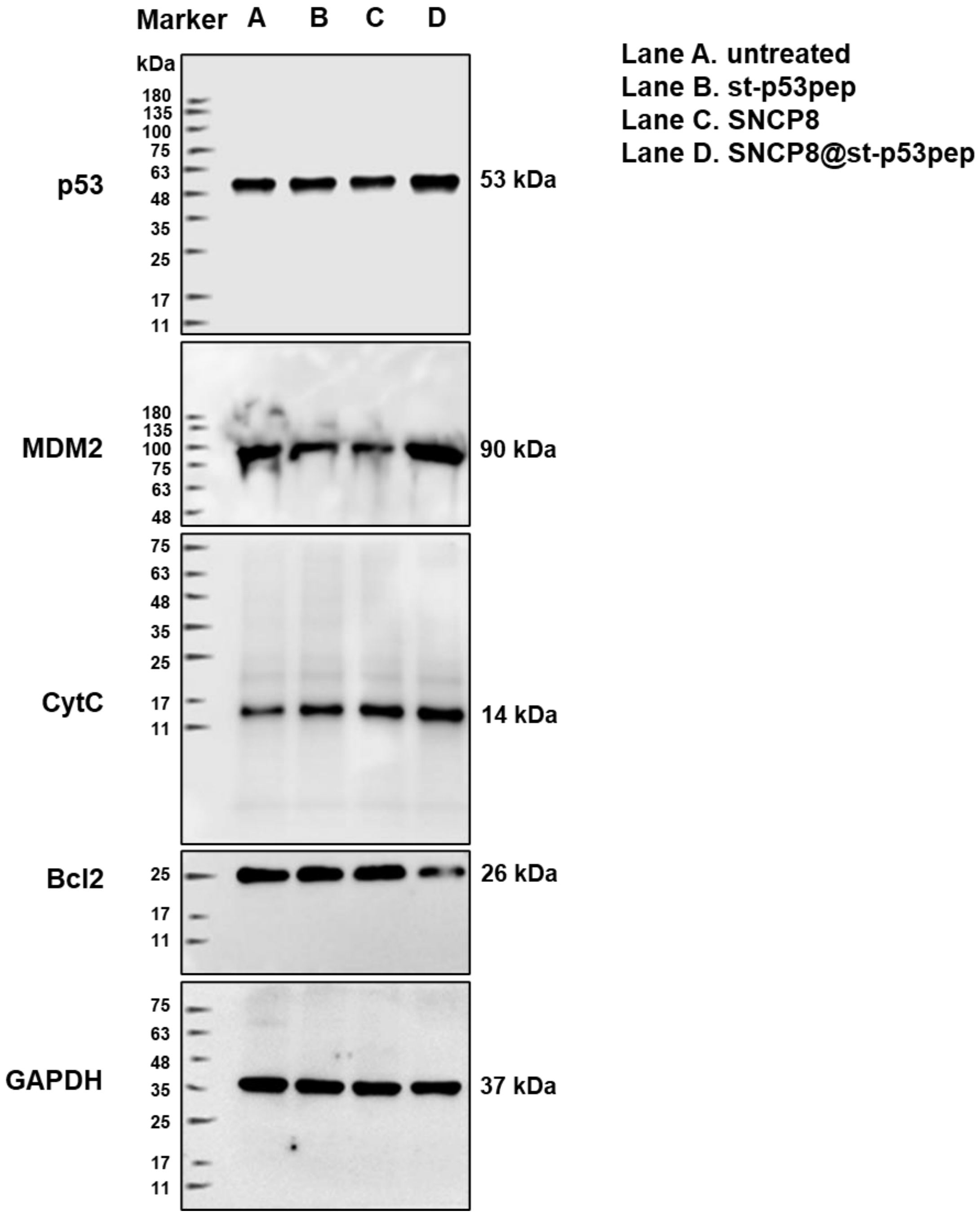

Supplementary Figure 18. Western blotting for protein expression involved in p53-mediated apoptosis pathway. Apoptosis related protein expression analysis in HepG2 cells after treating SNCP8@st-p53pep, st-p53pep and SNCP8. Protein levels of p53, MDM2, CytC, Bcl2 and GAPDH were determined by Western blot.

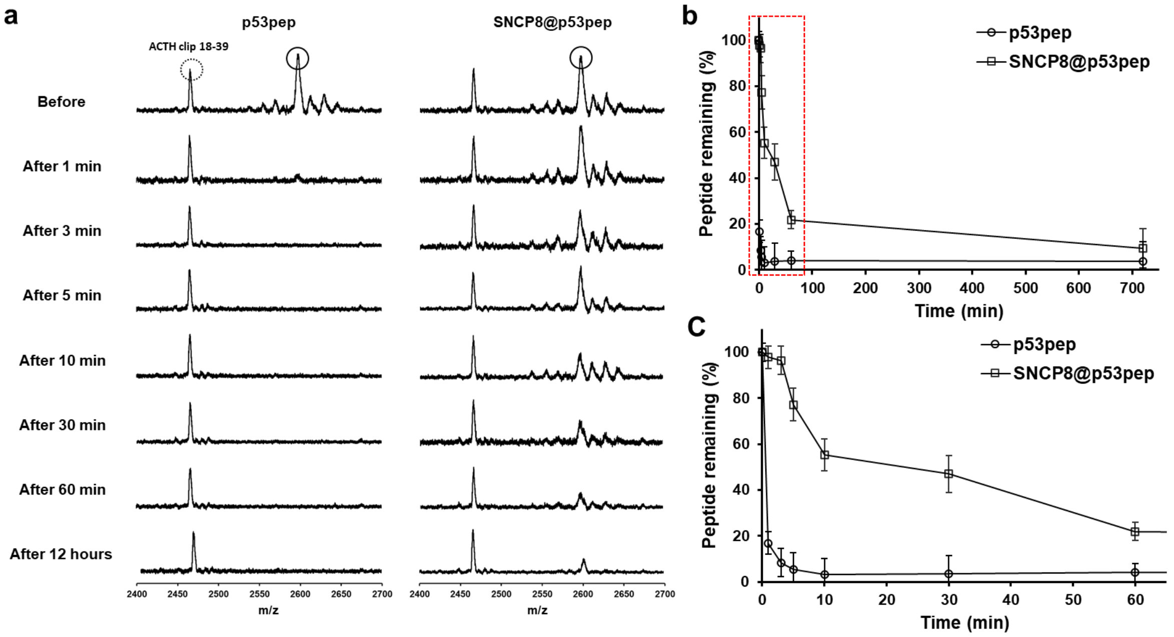

Supplementary Figure 19. The proteolytic degradation of p53pep. Semi-quantitative analysis of protease-mediated degradation of p53pep by using the matrix-assisted laser desorption ionization time-of-flight mass spectrometry (MALDI-TOF MS). (a) Mass spectrum of p53pep without SNCP8 (left) and with SNCP8 (right) over 12 h. The cumulative remaining p53pep in short (b) and long (c) periods in different conditions: p53pep (circle) and SNCP8@p53pep (square). Rapid degradation of free p53pep within 1 min is observed while the degradation of p53pep inside the SNCP8 is considerably slow and possessed the long-term stability even in the presence of proteinase K (N = 3, biologically independent samples; mean ± SD).

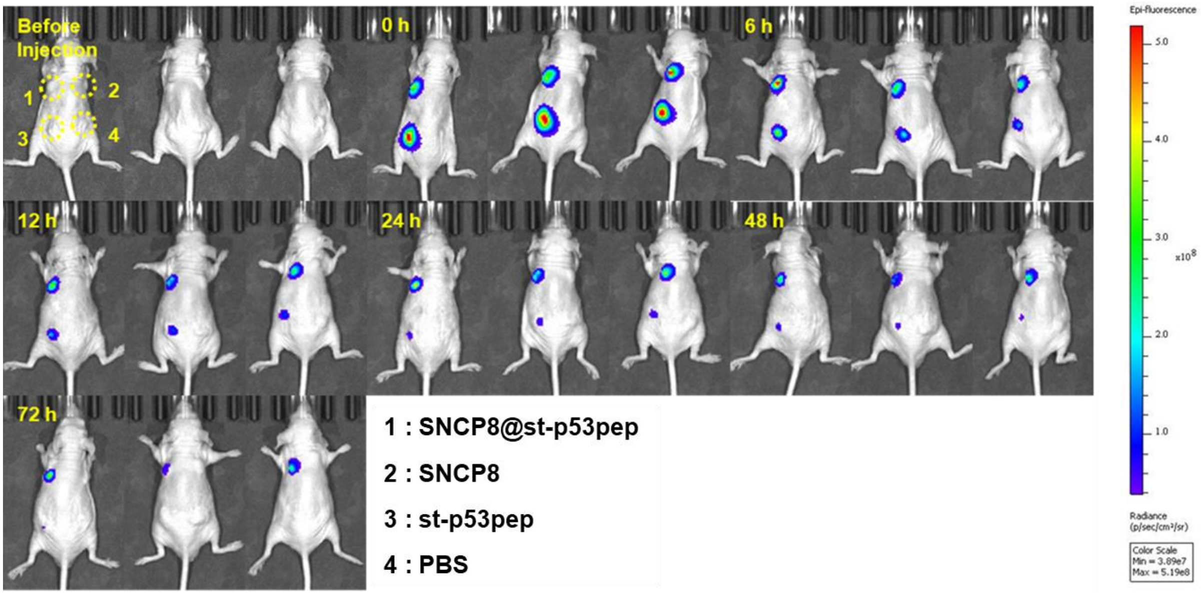

Supplementary Figure 20. The changes in fluorescence intensity corresponding to st-p53pep in mice. Time-dependent whole body fluorescence images of tumor-xenograft mice injected with SNCP8@st-p53pep and its comparative groups. (Fluorescence signal: st-p53pep; N = 3, biologically independent animals)
