# Supplementary material for: Multifunctional synthetic nano-chaperone for peptide folding and intracellular delivery
Source: Nat Commun. 2022 Aug 5;13:4568. doi: 10.1038/s41467-022-32268-2 (PMC9356039; doi:10.1038/s41467-022-32268-2)
Supplement: Supplementary file 2 — Reporting Summary [file 41467_2022_32268_MOESM2_ESM.pdf]

nature portfolio

Corresponding author(s): Dal-Hee Min

Last updated by author(s): Jul 21, 2022

Reporting Summary

Nature Portfolio wishes to improve the reproducibility of the work that we publish. This form provides structure for consistency and transparency in reporting. For further information on Nature Portfolio policies, see our Editorial Policies and the Editorial Policy Checklist.

Statistics

For all statistical analyses, confirm that the following items are present in the figure legend, table legend, main text, or Methods section.

n/a	Confirmed	
☐	☒	The exact sample size (*n*) for each experimental group/condition, given as a discrete number and unit of measurement
☐	☒	A statement on whether measurements were taken from distinct samples or whether the same sample was measured repeatedly
☐	☒	The statistical test(s) used AND whether they are one- or two-sided<br>*Only common tests should be described solely by name; describe more complex techniques in the Methods section.*
☒	☐	A description of all covariates tested
☒	☐	A description of any assumptions or corrections, such as tests of normality and adjustment for multiple comparisons
☐	☒	A full description of the statistical parameters including central tendency (e.g. means) or other basic estimates (e.g. regression coefficient) AND variation (e.g. standard deviation) or associated estimates of uncertainty (e.g. confidence intervals)
☐	☒	For null hypothesis testing, the test statistic (e.g. F, t, r) with confidence intervals, effect sizes, degrees of freedom and P value noted<br>*Give P values as exact values whenever suitable.*
☒	☐	For Bayesian analysis, information on the choice of priors and Markov chain Monte Carlo settings
☒	☐	For hierarchical and complex designs, identification of the appropriate level for tests and full reporting of outcomes
☒	☐	Estimates of effect sizes (e.g. Cohen's *d*, Pearson's *r*), indicating how they were calculated

Our web collection on statistics for biologists contains articles on many of the points above.

Software and code

Policy information about availability of computer code

Data collection

J-810 spectropolarimeter (JASCO, Japan)
UV-2550 spectrophotometer (Shimadzu, Japan)
SynergyMx (Biotek, UK)
spectrofluorometer FP-8300 (Jasco, USA)
Hyperion 2000 microscope optically coupled to a Bruker Optics Vertex 70 (Bruker, USA)
Energy-filtering transmission electron microscope (EF-TEM) LIBRA 120 (Carl Zeiss, Germany)
DeltaVision Elite Microscopy System (GE Healthcare, USA)
ImageQuantTM LAS 4000 mini (GE Healthcare, USA)
FoBI (Neoscience, Korea)
ultrafleXtreme MALDI-TOFTOF Spectrometer(Bruker, Germany)
Ultimate3000 Preparative HPLC system (Thermo Dionex, USA)
IVIS Spectrum In Vivo Imaging System (PerkinElmer, USA)

Data analysis

Microsoft Office Excel 2016, Power point 2016, Origin 2017, GraphPad Prism 7, Image J 1.52a
the CONTIN-LL algorithms provided from the DICHROWEB server (http://dichroweb.cryst.bbk.ac.uk/html/links.shtml)

For manuscripts utilizing custom algorithms or software that are central to the research but not yet described in published literature, software must be made available to editors and reviewers. We strongly encourage code deposition in a community repository (e.g. GitHub). See the Nature Portfolio guidelines for submitting code & software for further information.

Data

Policy information about availability of data

All manuscripts must include a data availability statement. This statement should provide the following information, where applicable:

- Accession codes, unique identifiers, or web links for publicly available datasets
- A description of any restrictions on data availability
- For clinical datasets or third party data, please ensure that the statement adheres to our policy

The authors declare that the data generated or analysed in this study are provided in the Supplementary Information/Source Data file. Data is available from the corresponding author upon request.

Human research participants

Policy information about studies involving human research participants and Sex and Gender in Research.

Reporting on sex and gender	not applicable
Population characteristics	not applicable
Recruitment	not applicable
Ethics oversight	not applicable

Note that full information on the approval of the study protocol must also be provided in the manuscript.

Field-specific reporting

Please select the one below that is the best fit for your research. If you are not sure, read the appropriate sections before making your selection.

[x] Life sciences [ ] Behavioural & social sciences [ ] Ecological, evolutionary & environmental sciences

For a reference copy of the document with all sections, see nature.com/documents/nr-reporting-summary-flat.pdf

Life sciences study design

All studies must disclose on these points even when the disclosure is negative.

Sample size	Sample size was chosen based on prior experience of the investigators with similar experiments previously published.
Data exclusions	No experimental data was excluded.
Replication	Experiments were repeated over triplicate. All attempts at replication were successful. Key data generated by one co-author were repeated by other co-authors.
Randomization	For the in vitro experiments, samples were randomly allocated into experimental groups. For the in vivo studies, animals were randomly grouped.
Blinding	The investigators were not blinded since the experimental group is clear in all experiments, as it is not an experiment that requires data to be hidden or a sample that requires encryption.

Reporting for specific materials, systems and methods

We require information from authors about some types of materials, experimental systems and methods used in many studies. Here, indicate whether each material, system or method listed is relevant to your study. If you are not sure if a list item applies to your research, read the appropriate section before selecting a response.

Materials & experimental systems

n/a	Involved in the study	
☐	☒	Antibodies
☐	☒	Eukaryotic cell lines
☒	☐	Palaeontology and archaeology
☐	☒	Animals and other organisms
☒	☐	Clinical data
☒	☐	Dual use research of concern

Methods

n/a	Involved in the study	
☒	☐	ChIP-seq
☒	☐	Flow cytometry
☒	☐	MRI-based neuroimaging

Antibodies

Antibodies used	Antibodies used for western blotting:<br>GAPDH rabbit pAb (Abcam, ab9485, USA), 1:2000<br>p53 (7F5) rabbit mAb (Cell Signaling Technology, 2527S, USA), 1:1000<br>MDM2 (D1V2Z) rabbit mAb (Cell Signaling Technology, 86934S, USA), 1:1000<br>Bcl2 (D55G8) rabbit mAb (Cell Signaling Technology, 4223S, USA), 1:1000<br>CytC (D18C7) rabbit mAb (Cell Signaling Technology, 11940S, USA), 1:1000<br>Anti-rabbit goat pAb, HRP-linked Antibody (Abcam, ab205718, USA),1:5000
Validation	Antibodies were validated for each application by using the manufacturer's guidelines. Multiple dilutions were tested to determine the most appropriate dilution. Manufacturers released certificates of analysis for each lot used.<br>All used antibodies have been validated by western blot.<br>GAPDH mAb (Abcam, ab9485, USA) (https://www.abcam.com/gapdh-antibody-loading-control-ab9485.html)<br>p53 (7F5) Rabbit mAb (Cell Signaling Technology, 2527S, USA) (https://www.cellsignal.com/products/primary-antibodies/p53-7f5-rabbit-mab/2527)<br>MDM2 (D1V2Z) Rabbit mAb (Cell Signaling Technology, 86934S, USA) (https://www.cellsignal.com/products/primary-antibodies/mdm2-d1v2z-rabbit-mab/86934)<br>Bcl2 (D55G8) Rabbit mAb (Cell Signaling Technology, 4223S, USA) (https://www.cellsignal.com/products/primary-antibodies/bcl-2-124-mouse-mab/15071)<br>CytC (D18C7) Rabbit mAb (Cell Signaling Technology, 11940S, USA) (https://www.cellsignal.com/products/primary-antibodies/cytochrome-c-d18c7-rabbit-mab/11940)<br>Anti-rabbit goat pAb, HRP-linked Antibody (Abcam, ab205718, USA) (https://www.abcam.com/goat-rabbit-igg-hl-hrp-ab205718.html)

Eukaryotic cell lines

Policy information about cell lines and Sex and Gender in Research

Cell line source(s)	HeLa, HepG2 cell lines used in the study are from ATCC.
Authentication	None of the cell lines were authenticated.
Mycoplasma contamination	All cell lines tested negative for mycoplasma contamination.
Commonly misidentified lines (See ICLAC register)	No commonly misidentified cell lines were used.

Animals and other research organisms

Policy information about studies involving animals; ARRIVE guidelines recommended for reporting animal research, and Sex and Gender in Research

Laboratory animals	Five-week-old, male, 20 ± 1 grams, pathogen-free Balb/c nude mice were used. Mice were housed in an environmentally controlled room (23 °C, with 55 ± 5% humidity and 12 h / 12 h light–dark cycle).
Wild animals	The study did not involve wild animals.
Reporting on sex	Since sex differences were not important in this experiment, sex was not considered; thus, the experiment was conducted with males. The overall number of mice used is 31.
Field-collected samples	The study did not involve samples collected from the field.
Ethics oversight	The research protocol was approved by the Ethics Committee and this study was complied with the guidelines of the Institutional Animal Care and Use Committee (IACUC) of Seoul National University.

Note that full information on the approval of the study protocol must also be provided in the manuscript.
